# Neurofibromin 1 mediates sleep depth in *Drosophila*

**DOI:** 10.1371/journal.pgen.1011049

**Published:** 2023-12-13

**Authors:** Elizabeth B. Brown, Jiwei Zhang, Evan Lloyd, Elizabeth Lanzon, Valentina Botero, Seth Tomchik, Alex C. Keene

**Affiliations:** 1 Department of Biology, Texas A&M University, College Station, Texas, United States of America; 2 Department of Biological Sciences, Florida State University, Tallahassee, Florida, United States of America; 3 Jupiter Life Science Initiative, Florida Atlantic University, Jupiter, Florida, United States of America; 4 Department of Neuroscience and Pharmacology, University of Iowa Carver College of Medicine, Iowa City, Iowa, United States of America; HudsonAlpha Institute for Biotechnology, UNITED STATES

## Abstract

Neural regulation of sleep and metabolic homeostasis are critical in many aspects of human health. Despite extensive epidemiological evidence linking sleep dysregulation with obesity, diabetes, and metabolic syndrome, little is known about the neural and molecular basis for the integration of sleep and metabolic function. The RAS GTPase-activating gene *Neurofibromin* (*Nf1*) has been implicated in the regulation of sleep and metabolic rate, raising the possibility that it serves to integrate these processes, but the effects on sleep consolidation and physiology remain poorly understood. A key hallmark of sleep depth in mammals and flies is a reduction in metabolic rate during sleep. Here, we examine multiple measures of sleep quality to determine the effects of *Nf1* on sleep-dependent changes in arousal threshold and metabolic rate. Flies lacking *Nf1* fail to suppress metabolic rate during sleep, raising the possibility that loss of *Nf1* prevents flies from integrating sleep and metabolic state. Sleep of *Nf1* mutant flies is fragmented with a reduced arousal threshold in *Nf1* mutants, suggesting *Nf1* flies fail to enter deep sleep. The effects of *Nf1* on sleep can be localized to a subset of neurons expressing the GABA_A_ receptor *Rdl*. Sleep loss has been associated with changes in gut homeostasis in flies and mammals. Selective knockdown of *Nf1* in *Rdl*-expressing neurons within the nervous system increases gut permeability and reactive oxygen species (ROS) in the gut, raising the possibility that loss of sleep quality contributes to gut dysregulation. Together, these findings suggest *Nf1* acts in GABA-sensitive neurons to modulate sleep depth in *Drosophila*.

## Author summary

Growing evidence suggests fruit flies, like mammals, possess different forms of sleep including light and deep sleep. Despite major advances in our understanding of t genes regulating sleep duration, little is known about how different forms of sleep are regulated. *Neurofibramin 1* is associated with numerous neurological phenotypes including dysregulated sleep and circadian rhythms. Here, we report that flies harboring mutations in *Neurofibramin 1* fail to enter deep sleep. This phenotype can be localized to GABA-receptive neurons in the brain and selective loss of *Neurofibramin 1* in these neurons is also associated with reduced longevity and gut dysregulation. Together, these findings provide insight into the neural basis of sleep depth in fruit flies.

## Introduction

The functional and neural basis of sleep is highly conserved from invertebrates through mammals [1,2]. In many cases, powerful genetics in relatively simple model systems, including the fruit fly, *Drosophila melanogaster*, have allowed for the identification of novel genes and neural mechanisms that have informed our understanding of human sleep [3,4]. However, most work in these models have studied total sleep duration. Therefore, a lack of understanding of the mechanisms underlying sleep quality and broader changes in physiology associated with sleep in non-mammalian models represents a significant gap in our knowledge. In mammals, slow-wave sleep is associated with reduced metabolic rate [5–7]. Growing evidence suggests that many physiological changes associated with mammalian sleep are conserved in flies, including a reduction in whole body metabolic rate [8,9]. The diverse physiological changes associated with sleep, including changes in body temperature, reduced metabolic rate, and synaptic homeostasis, are thought to be critical for sleep’s rejuvenate properties [10–12].

Flies, like mammals, exhibit distinct electrophysiological patterns that correlate with wake and rest [13–15]. We have identified sleep-associated reductions in metabolic rate in flies that are consistent with those that occur in mammals [8,16]. In addition, flies display all the behavioral hallmarks of sleep, including an extended period of behavioral quiescence, rebound following deprivation, increased arousal threshold, and species-specific posture [17,18]. Behavioral tracking systems and software are available for high-throughput detection and analysis of fly sleep using infrared monitoring or video tracking [19,20]. Sleep in *Drosophila* is typically defined as 5 minutes or more of behavioral quiescence, as this correlates with other behavioral and physiological characteristics that define sleep [8,14,21]. For example, sleep bouts lasting ~10 minutes or longer are associated with increased arousal threshold and low-frequency oscillations in brain activity. These findings are supported by computational analysis modeling sleep pressure [14,21,22]. These analyses suggest the presence of light and deep sleep in flies; however, the genetic and neural basis for these different types of sleep is poorly understood.

The *Nf1* gene encodes a large protein that functions as a negative regulator of Ras signaling and mediates pleiotropic cellular and organismal function [23,24]. *NF1* mutations in humans cause a disorder called neurofibromatosis type 1, characterized by benign tumors of the nervous system (neurofibromas), as well as increased susceptibility to neurocognitive deficits (e.g., attention-deficit/hyperactivity disorder, autism spectrum disorder, visuospatial memory impairments;[23]. In addition, mutation of *Nf1* is associated with dysregulated sleep and circadian rhythms [25,26]. *Drosophila* deficient for *Nf1* recapitulate many of these phenotypes and are widely used as a model to investigate the role of *Nf1* in regulation of cellular and neural circuit function [27]. Furthermore, *Drosophila Nf1* mutations lead to dysregulated circadian function and shortened sleep [25]. Here, we examine the effects of *Nf1* on sleep-dependent changes in metabolic rate and measues of sleep depth.

We find that flies lacking *Nf1* fail to suppress metabolic rate during prolonged sleep bouts, revealing a disruption of sleep-dependent changes in metabolic rate. Furthermore, multiple behavioral measurements suggest sleep depth is disrupted in *Nf1* mutant flies, including the presence of sleep fragmentation and reduced arousal threshold. Genetic and pharmacological analysis suggest *Nf1* modulates GABA signaling to regulate sleep depth and sleep-dependent changes in metabolic rate. Therefore, these findings suggest that *Nf1* is a critical regulator of sleep-metabolism interactions, and the conserved molecular and phenotypic nature of *Nf1* mutants raises the possibility that these findings may be relevant to the complex pathologies in humans afflicted with NF1.

## Results

**Fig 1:**
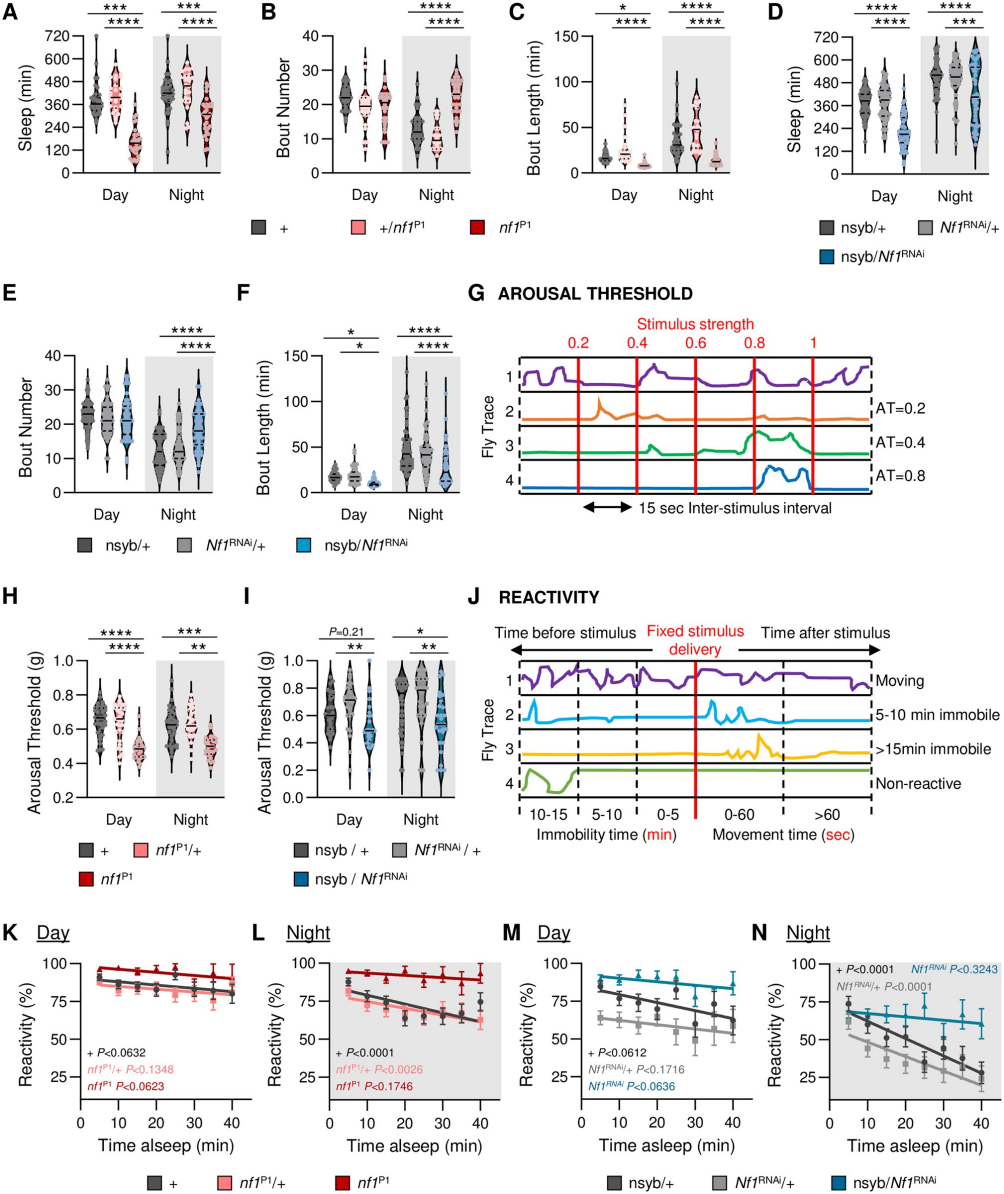
Loss of *Nf1* increases sleep fragmentation and decreases sleep depth. **(A-C)** Sleep traits of *nf1*^P1^ mutants, heterozygotes, and their respective control. **A**. There is a significant effect of genotype on sleep duration (two-way ANOVA: F_2,172_ = 80.70, *P*<0.0001). Compared to control and heterozygote flies, *nf1*^P1^ mutants sleep significantly less during the day (+, *P*<0.0001; het, *P*<0.0001) and night (+, *P*<0.0001; het, *P*<0.0001). **B.** There is a significant effect of genotype on bout number (two-way ANOVA: F_2,172_ = 21.15, *P*<0.0001). Compared to control and heterozygote flies, *nf1*^P1^ mutants have significantly more sleep bouts during the night (+, *P*<0.0001; het, *P*<0.0001), with no difference during the day (+, *P*<0.0512; het, *P*<0.0562). **C.** There is a significant effect of genotype on bout length (two-way ANOVA: F_2,172_ = 53.88, *P*<0.0001). Compared to control and heterozygote flies, *nf1*^P1^ mutants have significantly lower bout lengths during the day (+, *P*<0.0201; het, *P*<0.0001), and night (+, *P*<0.0001; het, *P*<0.0001). N = 26–32. **(D-F)** Sleep traits of pan-neuronal *Nf1*^RNAi^ knockdown flies and their respective controls.**D**. There is a significant effect of genotype on sleep duration (two-way ANOVA: F_2,312_ = 46.27, *P*<0.0001). Compared to controls, pan-neuronal knockdown of *Nf1* significantly reduces sleep during the day (nsyb/+, *P*<0.0001; *Nf1*^RNAi^/+, *P*<0.0001) and night (nsyb/+, *P*<0.0001; *Nf1*^RNAi^/+, *P*<0.0004). **E**. There is a significant effect of genotype on bout number (two-way ANOVA: F_2,312_ = 7.728, *P*<0.0005). Compared to controls, pan-neuronal knockdown of *Nf1* significantly increases bout number during the night (nsyb/+, *P*<0.0001; *Nf1*^RNAi^/+, *P*<0.0001), with no difference during the day (nsyb/+, *P*<0.6476; *Nf1*^RNAi^/+, *P*<0.9943). **F**. There is a significant effect of genotype on bout length (two-way ANOVA: F_2,312_ = 19.68, *P*<0.0001). Compared to controls, pan-neuronal knockdown of *Nf1* significantly reduces bout length during the day (nsyb/+, *P*<0.0178; *Nf1*^RNAi^/+, *P*<0.0253) and night (nsyb/+, *P*<0.0001; *Nf1*^RNAi^/+, *P*<0.0001). N = 50–55. **(G-N)**. The *Drosophila* Arousal Tracking (DART) was used to probe arousal threshold and reactivity measurements. This system records fly movement while simultaneously controlling mechanical stimuli via a digital analog converter (DAC). All measurements included were taken from sleeping flies and determined hourly, starting at ZT0. **G**. Mechanical stimuli of increasing strength were used to assess arousal threshold in all sleeping flies and was determined by fly movement within 15 sec of stimulus delivery. **H**. There is a significant effect of genotype on arousal threshold (REML: F_2,94_ = 37.62, *P*<0.0001; N = 29–35). Compared to control and heterozygote flies, arousal threshold significantly decreases in *nf1*^P1^ mutants and occurs during the day (+, *P*<0.0001; het, *P*<0.0001) and night (+, *P*<0.0001; het, *P*<0.0001). N = 30–35. **I**. There is a significant effect of genotype on arousal threshold (REML: F_2,98_ = 7.795, *P*<0.0007; N = 25–40). Compared to controls, pan-neuronal knockdown of *Nf1* has mixed effects on arousal threshold during the day (nsyb/+, *P*<0.2138; *Nf1*^RNAi^/+, *P*<0.0040) and significantly decreases arousal threshold during the night (nsyb/+, *P*<0.0271; *Nf1*^RNAi^/+, *P*<0.00062). N = 25–40. **J**. Mechanical stimuli at maximum intensity was used to measure reactivity as a function of time spent asleep. A fly was considered reactive if it moved within 60 sec of stimulus delivery. **K**. Linear regression of daytime reactivity as a function of time asleep in *nf1*^P1^ mutants, heterozygotes, and their respective control. The intercepts of each regression line are significantly different from each other (F_2,2064_ = 34.77, *P*<0.0001). **L**. Linear regression of nighttime reactivity as a function of time asleep in *nf1*^P1^ mutants, heterozygotes, and their respective control. The intercepts of each regression line are significantly different from each other (F_2,2102_ = 57.05, *P*<0.0001). N = 58–70. **M**. Linear regression of daytime reactivity as a function of time asleep in pan-neuronal *Nf1*^RNAi^ knockdown flies and their controls. The intercepts of each regression line are significantly different from each other (F_2,1287_ = 53.12, *P*<0.0001). **N**. Linear regression of nighttime reactivity as a function of time asleep in pan-neuronal *Nf1*^RNAi^ knockdown flies and their controls. The intercepts of each regression line are significantly different from each other (F_2,1564_ = 4.887, *P*<0.0077). N = 36–41. For sleep and arousal threshold measurements, the median (solid line), as well as the 25th and 75th percentiles (dotted lines) are shown. For reactivity, error bars indicate ± SEM. The *P*-values in each panel indicates whether the slope of the regression line is significantly different from zero. White background indicates daytime, while gray background indicates nighttime. *p<0.05; **p<0.01; ***p<0.001; ****p<0.0001.

To examine the effects of *Nf1* on sleep and activity, we compared sleep of control flies to *nf1*^P1^ mutants that harbor a near-total deletion in the *Nf1* locus [28]. Sleep was reduced during the day and night in *nf1*^P1^ mutants compared to controls (Fig 1A), consistent with previous literature [29,30]. Sleep duration in *nf1*^P1^ heterozygous flies did not differ from controls, indicating that the phenotype is recessive (Fig 1A). The average number of sleep bouts was increased during the night in *nf1*^P1^ flies, while the average bout length was reduced compared to control and heterozygote flies during both the day and night, suggesting that loss of *Nf1* results in sleep fragmentation (Fig 1B and 1C). In addition to the loss of sleep, the average velocity of activity during waking periods (waking activity) is elevated, suggesting that loss of *nf1*^P1^ also results in hyperactivity (S1A Fig). These findings suggest *Nf1* promotes sleep duration, consolidation of sleep bouts, and modulates waking activity.

To determine if the sleep and activity phenotypes of *Nf1* are due to loss of function in neurons, we selectively knocked down *Nf1* by expressing *Nf1*^RNAi^ under the control of the pan-neuronal driver nsyb-GAL4. Sleep was reduced and fragmented in flies upon pan-neuronal knockdown of *Nf1* in neurons (nsyb-GAL4/*Nf1*^RNAi^) compared to flies harboring either transgene alone (Fig 1D–1F). Waking activity was also elevated with neuron-specific knockdown of *Nf1* (S1B Fig). To validate that these differences were not due to off-target effects of RNAi, we next confirmed these findings using an independently derived RNAi line [31]. We again found that pan-neuronal knockdown of *Nf1* significantly decreased sleep duration, while sleep fragmentation during the night and waking activity increased significantly (S2A–S2D Fig). Therefore, pan-neuronal knockdown of *Nf1* fully recapitulates the mutant phenotype, suggesting that *Nf1* functions in neurons to regulate sleep.

To further investigate the role of *Nf1* on sleep consolidation, we analyzed activity patterns using a Markov model that predicts sleeping and waking propensity, indicators of sleep depth [22]. In both *nf1*^P1^ mutants and nsyb-GAL4/*Nf1*^RNAi^ flies, loss of *Nf1* increases the propensity to wake, while sleep propensity is reduced or remains unchanged S2E and S2F and S3 Figs). Therefore, the phenotypes of both pan-neuronal RNAi knockdown of *Nf1* and genetic mutants further support the notion that *Nf1* promotes sleep and prevents sleep fragmentation.

Mounting evidence suggests that flies, like mammals, possess distinct sleep stages comprised of light and deep sleep [21,32,33]. Increased arousal threshold, the phenomenon where a stronger stimulus is required to induce movement, is a key hallmark of sleep that is conserved across phyla [34]. Longer nighttime sleep bouts are associated with elevated arousal threshold, suggesting that sleep intensity increases during longer sleep bouts [32,34]. To determine whether sleep depth is disrupted in *Nf1* deficient flies, we used the *Drosophila Arousal Tracking* (DART) system. We first implemented a paradigm that provides sleeping flies with increasing levels of vibration stimuli to determine the magnitude of the stimulus required to awaken the fly, a metric that is independent of time spent asleep (Fig 1G; [32] Arousal threshold was reduced during the day and the night in *nf1*^P1^ mutant flies compared to wild-type controls and heterozygotes, revealing reduced sleep depth associated with loss of *Nf1* (Fig 1H). Similarly, arousal threshold was reduced during the day and night in flies with pan-neuronal knockdown of *Nf1* (nsyb-GAL4/*Nf1*^RNAI^) compared to flies harboring either transgene alone (Fig 1I). Sleep duration was also reduced in *nf1*^P1^ mutants and upon pan-neuronal knockdown of *Nf1* in this system (S4A and S4B Fig). Together, these findings reveal that neuronal *Nf1* is required for normal arousal threshold.

To determine whether loss of *Nf1* impacts the propensity of stimuli to awaken flies across individual sleep bouts, we measured the reactivity of flies to a fixed vibration stimulus and calculated their responsiveness as a function of time spent asleep prior to stimulus onset (Fig 1J). In control and heterozygous n*f1*^P1^ flies, there was no effect of time spent asleep on reactivity during the day (Fig 1K). However, reactivity was significantly reduced during the night, as the slope of their respective regression lines differed significantly from zero (Fig 1L). *nf1*^P1^ mutants had no effect on time spent asleep during the night or the day on reactivity (Fig 1K and 1L). Flies with pan-neuronal knockdown of *Nf1* maintained high levels of reactivity across sleep bouts of up to 40 minutes, phenocopying *nf1*^P1^ mutants (Fig 1M and 1N). Therefore, *Nf1* is required for sleep duration-dependent changes in arousal threshold.

**Fig 2:**
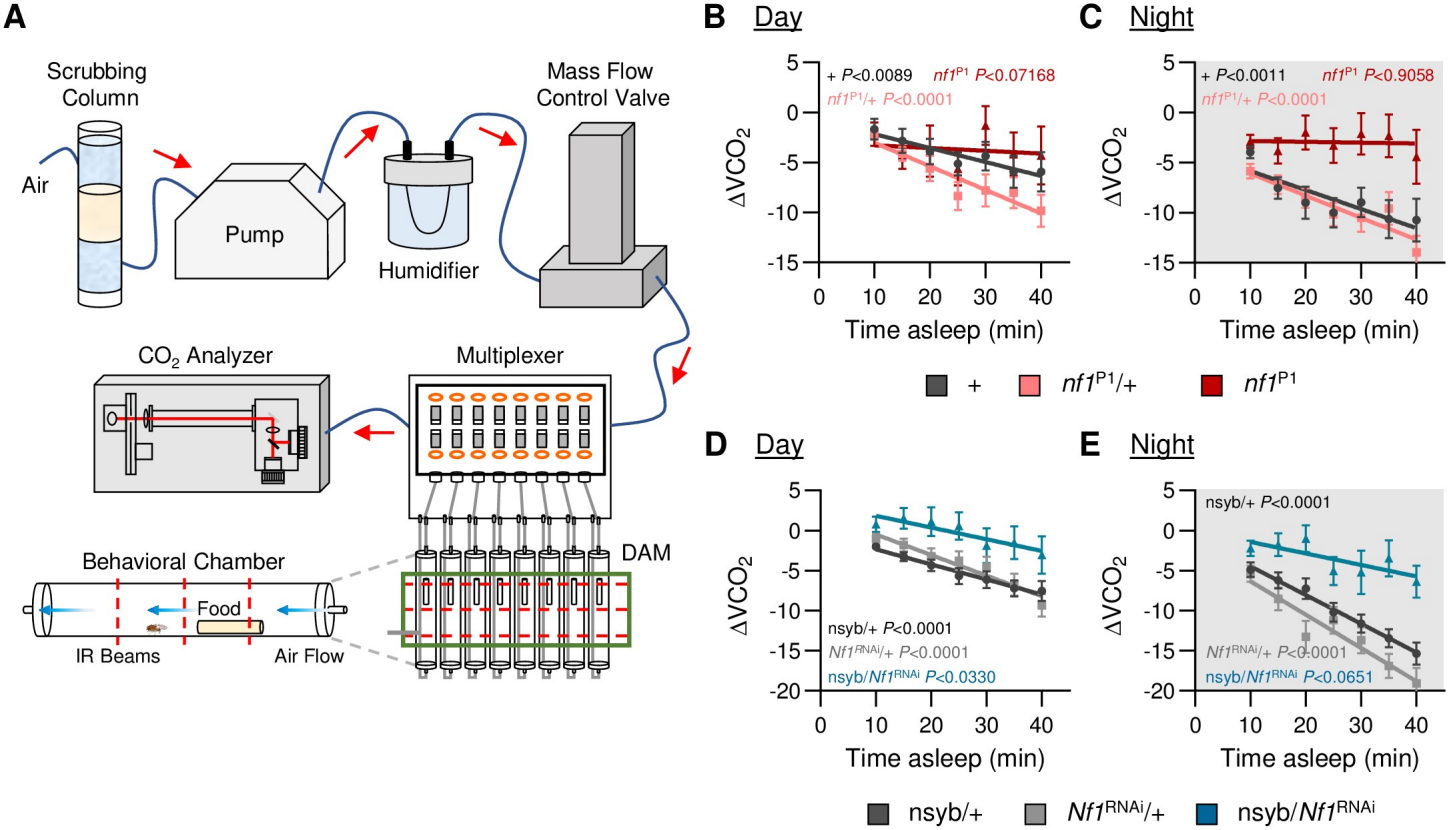
Loss of *Nf1* disrupts metabolic regulation of sleep. The Sleep and Metabolic Monitoring (SAMM) system was used to measure metabolic rate as a function of time spent asleep. **A**. Overview of the SAMM system. This system records activity while simultaneously measuring CO_2_ production, thereby enabling sleep and activity metrics to be paired with CO_2_ output. **B**. Linear regression of daytime CO_2_ output as a function of time asleep in *nf1*^P1^ mutants, heterozygotes, and their respective control. There is no significant difference between the slopes of each regression line (ANCOVA with time asleep as the covariate: F_2,532_ = 2.988, *P*<0.0512). N = 26–27. **C**. Linear regression of nighttime CO_2_ output as a function of time asleep in *nf1*^P1^ mutants, heterozygotes, and their respective control. The slopes of each regression line are significantly different from each other (F_2,538_ = 3.847, *P*<0.0219). **D**. Linear regression of daytime CO_2_ output as a function of time asleep in pan-neuronal *Nf1*^RNAi^ knockdown flies and their controls. There is no significant difference between the slopes of each regression line (F_2,805_ = 1.001, *P*<0.3680). **E**. Linear regression of nighttime CO_2_ output as a function of time asleep in pan-neuronal *Nf1*^RNAi^ knockdown flies and their controls. The slopes of each regression line are significantly different from each other (F_2,822_ = 5.625, *P*<0.0037). N = 30–44. Error bars indicate ± SEM. The *P*-values in each panel indicates whether the slope of the regression line is significantly different from zero. White background indicates daytime, while gray background indicates nighttime. *p<0.05; **p<0.01; ****p<0.0001.

In both flies and mammals, sleep is associated with reduced metabolic rate [8,9,35–39]. To determine the effect of *Nf1* on sleep-dependent modulation of metabolic rate, we measured metabolic rate in awake and sleeping flies using the Sleep and Activity Metabolic Monitor (SAMM) system. This system uses indirect calorimetry to measure CO_2_ release, while simultaneously measuring activity via counting infrared beam crosses (Fig 2A; In agreement with previous findings, sleep was reduced in *nf1*^P1^ mutants in the SAMM system, and the total metabolic rate (VCO_2_) was elevated during the day and the night compared to controls (S5A and S5B Fig; [40]). Similar effects were observed upon pan-neuronal knockdown of *Nf1* (S5C and S5D Fig). To specifically examine the effects of sleep on CO_2_ output, we compared the overall CO_2_ output during waking and sleep. We found that CO_2_ output was significantly higher in *Nf1* mutant flies during both waking and sleeping, and was consistent during the day and night (S6A and S6B Fig). Pan-neuronal knockdown of *Nf1* (nsyb-GAL4/*Nf1*^RNAi^) similarly resulted in significantly higher CO_2_ output during both waking and sleeping (S6C and S6D Fig). This systematic dissection of CO_2_ output into sleep/waking states suggests that *Nf1* is required for the maintenance of metabolic rate.

To directly test whether sleep-metabolism interactions are disrupted by the loss of *Nf1*, we measured CO_2_ output over the length of a sleep bout. Metabolic rate was reduced during longer sleep bouts in control flies during the night, but did not change in *nf1*^P1^ mutants, while there was no effect of sleep on metabolic rate during the day (Fig 2B and 2C). Similarly, pan-neuronal knockdown of *Nf1* (nsyb-GAL4/*Nf1*^RNAi^) abolished nighttime sleep-dependent changes in metabolic rate, while there was no effect of sleep on metabolic rate during the day (Fig 2D and 2E). These findings reveal a critical role for neuronal *Nf1* in sleep-dependent changes in metabolic rate. Taken together, loss of *Nf1* results in sleep fragmentation, reduced arousal threshold, and loss of sleep-dependent changes in metabolic rate, suggesting that *Nf1* is required for flies to enter deep sleep.

**Fig 3:**
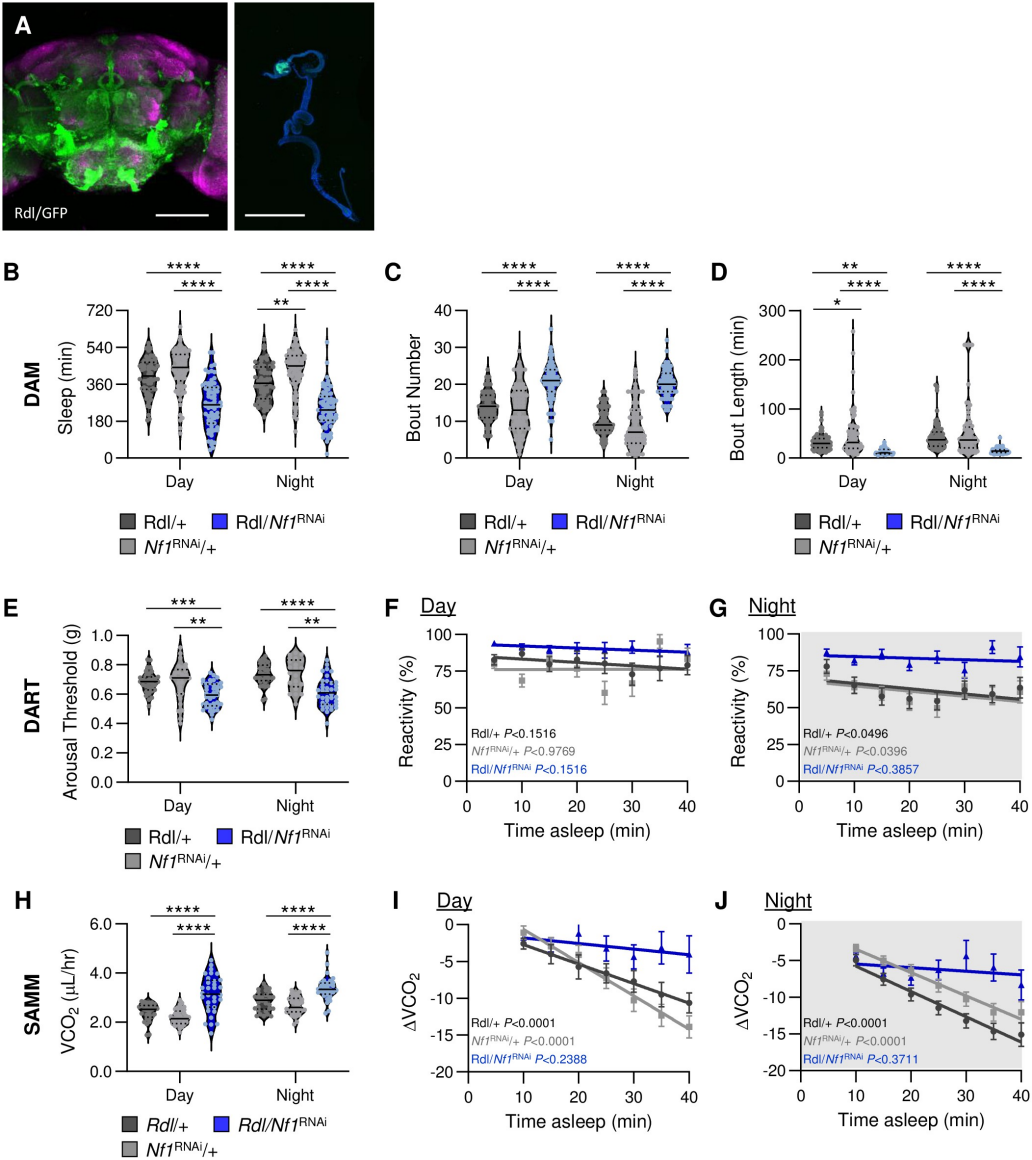
GABA_A_ receptor neurons mediate sleep depth via *Nf1*. GABA_A_ receptor neurons were targeted using the *Rdl*-GAL4 driver. **A**. The expression pattern of *Rdl*-expressing neurons is visualized with GFP in the brain (left) and the gut (right). For the brain, background staining is NC82 antibody (magenta); scale bar = 100μm. For the gut, background staining is DAPI (blue); scale bar = 1000μm. (**B-D**). Sleep traits of *Nf1*^RNAi^ knockdown flies and their respective controls. **B**. There is a significant effect of genotype on sleep duration (two-way ANOVA: F_2,362_ = 110.4, *P*<0.0001). Compared to controls, knockdown of *Nf1* in *Rdl*-expressing neurons significantly reduces sleep and occurs during the day (*Rdl*/+, *P*<0.0001; *Nf1*^RNAi^/+, *P*<0.0001) and night (*Rdl*/+, *P*<0.0001; *Nf1*^RNAi^/+, *P*<0.0001). **C**. There is a significant effect of genotype on bout number (two-way ANOVA: F_2,362_ = 115.6, *P*<0.0001). Compared to controls, knockdown of *Nf1* in *Rdl*-expressing neurons significantly increases bout number and occurs during the day (*Rdl*/+, *P*<0.0001; *Nf1*^RNAi^/+, *P*<0.0001) and night (*Rdl*/+, *P*<0.0001; *Nf1*^RNAi^/+, *P*<0.0001). **D**. There is a significant effect of genotype on bout length (two-way ANOVA: F_2,362_ = 37.97, *P*<0.0001). Compared to controls, knockdown of *Nf1* in *Rdl*-expressing neurons significantly reduces bout length and occurs during the day (*Rdl*/+, *P*<0.0068; *Nf1*^RNAi^/+, *P*<0.0001) and night (*Rdl*/+, *P*<0.0001; *Nf1*^RNAi^/+, *P*<0.0001). N = 57–66. **(E-G)** Measurements of arousal threshold and reactivity in *Nf1*^RNAi^ knockdown flies and their respective controls using the DART system. **E**. There is a significant effect of genotype on arousal threshold (REML: F_2,103_ = 13.76, *P*<0.0001; N = 22–38). Compared to controls, knockdown of *Nf1* in *Rdl*-expressing neurons significantly decreases arousal threshold and occurs during the day (*Rdl*/+, *P*<0.0003; *Nf1*^RNAi^/+, *P*<0.0011) and night (*Rdl*/+, *P*<0.0001; *Nf1*^RNAi^/+, *P*<0.0014). N = 29–41. **F**. Linear regression of daytime reactivity as a function of time asleep in *Nf1*^RNAi^ knockdown flies and their controls. The intercepts of each regression line are significantly different from each other (F_2,1771_ = 44.12, *P*<0.0001). **G**. Linear regression of nighttime reactivity as a function of time asleep in *Nf1*^RNAi^ knockdown flies and their controls. The intercepts of each regression line are significantly different from each other (F_2,1948_ = 64.06, *P*<0.0001). N = 49–61. **(H-J)** Measurements of metabolic rate in *Nf1*^RNAi^ knockdown flies and their respective controls using the SAMM system. **H**. There is a significant effect of genotype on metabolic rate (two-way ANOVA: F_2,188_ = 55.60, *P*<0.0001). Compared to controls, knockdown of *Nf1* in *Rdl*-expressing neurons significantly increases CO_2_ output and occurs during the day (*Rdl*/+, *P*<0.0001; *Nf1*^RNAi^/+, *P*<0.0001) and night (*Rdl*/+, *P*<0.0001; *Nf1*^RNAi^/+, *P*<0.0001). **I**. Linear regression of daytime CO_2_ output as a function of time asleep in pan-neuronal *Nf1*^RNAi^ knockdown flies and their controls. The slopes of each regression line are significantly different from each other (F_2,698_ = 11.17, *P*<0.0001). **J**. Linear regression of nighttime CO_2_ output as a function of time asleep in pan-neuronal *Nf1*^RNAi^ knockdown flies and their controls. The slopes of each regression line are significantly different from each other (F_2,756_ = 13.64, *P*<0.0001). N = 30–35. For violin plots, the median (solid line) as well as 25th and 75th percentiles (dotted lines) are shown. For reactivity and metabolic rate measurements, error bars indicate ± SEM. The *P*-values in each panel indicate whether the slope of the regression line is significantly different from zero. White background indicates daytime, while gray background indicates nighttime. *p<0.05; **p<0.01; ***p<0.001; ****p<0.0001.

While *Nf1* is broadly expressed throughout the brain, its function has been linked to the modulation of GABA signaling during the formation of associative memories [41]. In *Drosophila*, the GABA_A_ receptor *Resistant to dieldrin* (*Rdl)* is expressed in numerous populations of sleep-regulating neurons in the brain as well as in the ampula of the intestine (Fig 3A; [42–44]. To examine whether *Nf1* functions in GABA_A_ receptor neurons, we selectively knocked down *Nf1* by expressing *Nf1*^RNAi^ under the control of *Rdl*-GAL4 and then measured its effect on sleep. Flies with *Nf1* knockdown in GABA_A_ receptor neurons (*Rdl*-GAL4/*Nf1*^RNAi^) slept less than control flies harboring either transgene alone (Fig 3B). Sleep was fragmented in *Rdl*-GAL4*/Nf1*^*RNAi*^ flies, with increased bout number, reduced bout length, and an increased propensity to wake (Figs 3C and 3D and S7). Further, when sleep was measured in the DART and SAMM systems, knockdown of *Nf1* in GABA_A_ receptor neurons similarly reduced sleep duration (S8A and S8B Fig).

To verify that Rdl functions in the brain, we knocked down *Nf1* in flies that also contained the *tsh*-GAL80 transgene, that blocks GAL4 activity in the enteric nervous system [45,46]. The inclusion of *tsh*-GAL80 removed GFP-reporter expression in the gut, but left expression of Rdl-GAL4 in the central brain (S9A and S9B Fig). Reactivity during sleep long nighttime sleep bouts remained elevated in tsh-GAL80;Rdl-GAL4*/Nf1*^*RNAi*^ flies, supporting the notion that the phenotype observed in flies with Rdl knockdown is not derived by loss of *Nf1* in the gut (S9C and S9D Fig). Further, the expression pattern of Rdl-GAL4 was similar in control flies and those harboring *Nf1*^*RNAi*^ suggesting the UAS-*Nf1*^*RNAi*^ transgene is being faithfully expressed in Rdl-GAL4 positive neurons (S10 Fig). Therefore, knockdown of *Nf1* in GABA_A_ receptor neurons of the brain phenocopies pan-neuronal knockdown of *Nf1*, suggesting that GABA-sensitive neurons contribute to the sleep abnormalities of *Nf1* mutant flies.

It is possible that the effects on sleep duration and sleep depth are regulated by shared or distinct populations of neurons. Therefore, we sought to determine whether loss of *Nf1* in GABA_A_ receptor neurons also impacts arousal threshold and sleep-dependent changes in metabolic rate. Similar to pan-neuronal knockdown, arousal threshold was reduced in *Rdl*-GAL4/*Nf1*^RNAi^ flies, and these flies do not decrease reactivity during long nighttime sleep bouts (Fig 3E–3G). The metabolic phenotypes of *Nf1* mutant and pan-neuronal knockdown flies were also present in flies upon knockdown of *Nf1* in GABA_A_ receptor neurons. First, total metabolic rate was significantly increased, phenocopying pan-neuronal loss of *Nf1* (Fig 3H). Knockdown of *Nf1* in GABA_A_ receptor neurons similarly resulted in significantly higher CO_2_ output during both waking and sleeping states (S8C and S8D Fig). In addition, knockdown of *Nf1* in GABA_A_ receptor neurons abolished sleep-dependent changes in metabolic rate during the day and night (Fig 3I and 3J). Therefore, *Nf1* is required in GABA_A_ neurons to regulate sleep duration, arousal threshold, and sleep-dependent changes in metabolic rate.

**Fig 4:**
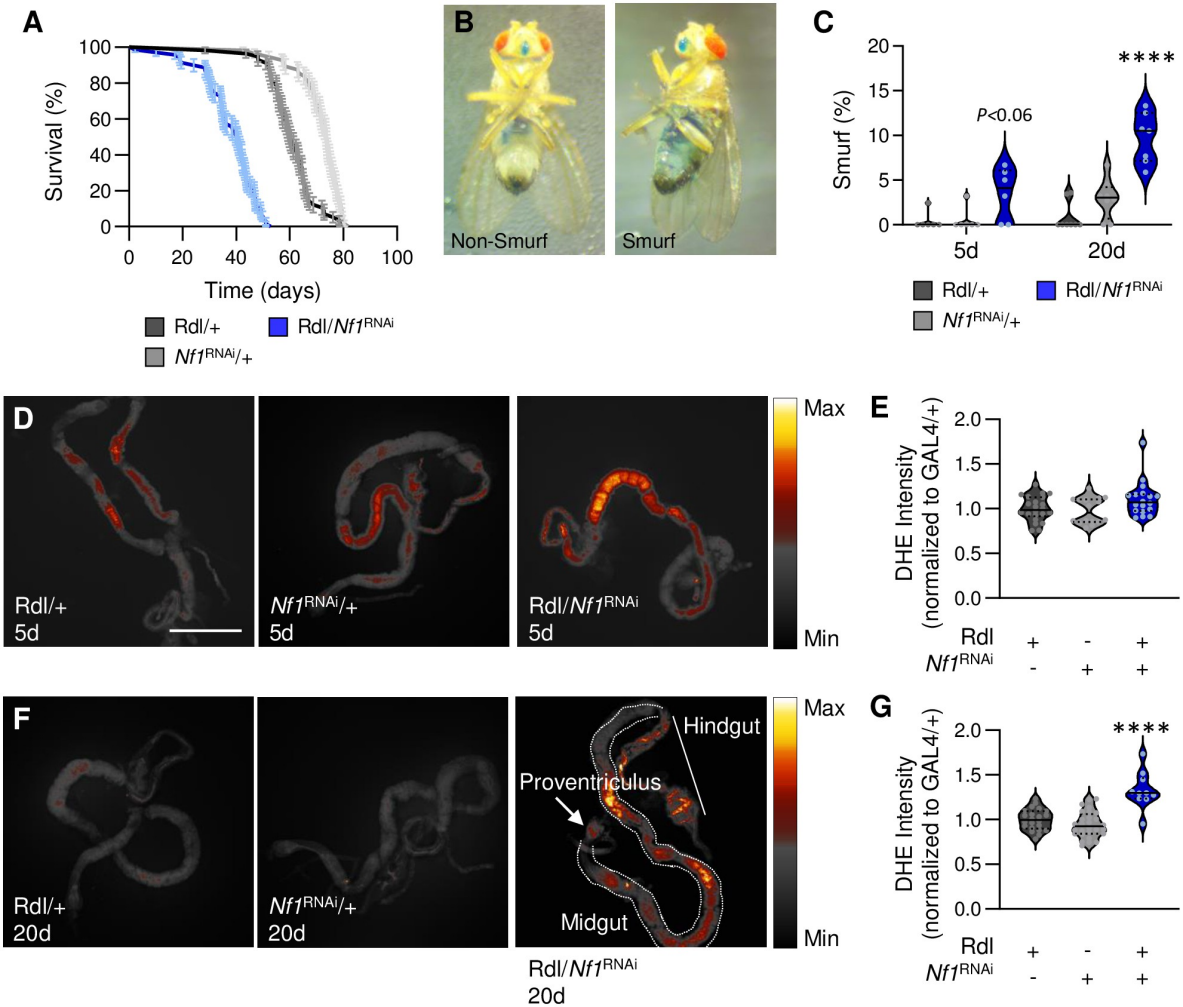
Loss of *Nf1* in GABA_A_ receptor neurons reduces longevity and promotes aging-associated phenotypes. **A**. Compared to controls, knockdown of *Nf1* in *Rdl*-expressing neurons significantly decreases longevity (Log-Rank test: χ^2^ = 253.4, d.f. = 2, *P*<0.0001). N = 57–70. **(B,C)** The Smurf assay was used to measure intestinal barrier dysfunction. **B.** Representative images depicting non-Smurf (left) and Smurf flies (right). **C.** There is a significant effect of genotype on intestinal permeability (two-way ANOVA: F_2,35_ = 29.45, *P*<0.0001). Knockdown of *Nf1* in *Rdl*-expressing neurons does not change intestinal barrier dysfunction in 5d flies (*Rdl*/+, *P*<0.0565; *Nf1*^RNAi^/+, *P*<0.0648), but significantly increases in 20d flies (*Rdl*/+, *P*<0.0001; *Nf1*^RNAi^/+, *P*<0.0001). N = 6–8. **(D-F)** ROS was measured in 5d and 20d flies by quantifying oxidized DHE. Scale bar = 500μm. **D** Oxidized DHE was measured in 5d control and *Nf1* knockdown flies. **E**. There is no significant difference in oxidized DHE signal intensity in 5d flies (one-way ANOVA: F_2,53_ = 3.367, *P*<0.0520). N = 16–22. **F**. Oxidized DHE was measured in 20d control and *Nf1* knockdown flies. **G**. Knockdown of *Nf1* in *Rdl*-expressing neurons significantly increases oxidized DHE signal intensity in 20d flies (one-way ANOVA: F_2,57_ = 25.76, *P*<0.0001). N = 10–28. The median (solid line) as well as 25th and 75th percentiles (dotted lines) are shown. ****p<0.0001.

In *Drosophila*, sleep loss has been associated with shortened lifespan [47–49]. However, a number of sleep mutants do not impact lifespan, and flies are able to survive prolonged periods of sleep deprivation [50–52]. To examine whether the disrupted sleep of *Nf1*-defficient flies impacts their lifespan, we measured the effects of loss of *Nf1* on longevity in individually housed flies. Lifespan was significantly reduced in *nf1*^P1^ mutant flies, as well as pan-neuronal knockdown (nsyb-GAL4/*Nf1*^RNAi^) or GABA_A_-receptor specific knockdown (*Rdl*-GAL4/*Nf1*^RNAi^) of *Nf1*, compared to their respective controls (Figs 4A and S11A and S11B). These findings suggest that the loss of *Nf1* affects sleep duration and sleep quality and results in a significantly reduced lifespan.

We next sought to measure the functional consequences of loss of *Nf1*. In *Drosophila* and mammals, chronic sleep loss is associated with deficiencies in gut homeostasis, that can result in death [49]. To measure gut integrity, flies were fed blue dye and then assayed for gut permeability [53,54]. In control flies, gut permeability remains intact in young (5 days) and aged (20 days) flies (Fig 4B and 4C). However, in *nf1*^P1^ mutants and flies with pan-neuronal knockdown of *Nf1*, gut permeability significantly increased in aged flies compared to controls (S11C and S11D Fig). Similarly, knockdown of *Nf1* in GABA_A_ receptor neurons (*Rdl*-GAL4/*Nf1*^RNAi^) significantly increased intestinal permeability in aged flies (Fig 4C). Together, these findings reveal that neuronal loss of *Nf1*, as well as selective loss in GABA_A_ receptor neurons, results in increased gut permeability that has been associated with aging.

It has previously been reported that chronic sleep deprivation induces the generation of reactive oxygen species (ROS) that underlies reduced lifespan, and this can be rescued by antioxidant feeding [49]. These findings suggest that low sleep quality negatively impacts the health and longevity of *Drosophila*. To determine if loss of *Nf1* impairs gut function, we measured ROS levels in the gut in young (5 days) and aged (20 days) flies. Pan-neuronal knockdown of *Nf1* (nsyb-GAL4/*Nf1*^RNAi^) led to increased ROS levels in both young and aged flies compared to controls (S12 Fig). Furthermore, ROS levels were also elevated in aged flies upon knockdown of *Nf1* in GABA_A_ receptor neurons (Fig 4D–4G). To test whether antioxidant feeding in *Nf1*^RNAi^ flies would promote survival and restore gut homeostasis, we added either Lipoic Acid or Melatonin to standard *Drosophila* media and then measured longevity and ROS in the gut. We found that antioxidant feeding had no effect on longevity, intestinal permeability, or ROS in Rdl-GAL4/*Nf1*^RNAi^ flies (S13 Fig). These findings suggest that under the conditions we used there is no effect of antioxidants on ROS and longevity. Overall, these findings support the notion that reduced gut function contributes to the reduced lifespan associated with the loss of *Nf1* and suggests that *Nf1* may act upstream of the protective effects of these antioxidants on lifespan and gut function.

Given the connection between decreased sleep quality and aging traits, we tested the hypothesis that sleep induction in *Nf1*^RNAi^ flies would promote survival and restore gut homeostasis. To pharmacologically induce sleep, we added Gaboxadol, a GABA_A_ Receptor agonist that has been widely used to increases sleep in *Drosophila* [34,55,56], to the standard fly media and then measured sleep depth and aging traits. We found that, similar to control flies, feeding Rdl-GAL4/*Nf1*^RNAi^ flies Gaboxadol increased both daytime and nighttime sleep, but was unable to restore arousal threshold (S14A–S14D Fig). Interestingly, Gaboxadol feeding across the lifespan extended longevity in control flies, but had no effect on Rdl-GAL4/*Nf1*^RNAi^ flies (S14E–S14G Fig). Gaboxadol feeding also had no effect on intestinal permeability or ROS (S14H–S14J Fig). These results support the notion that the reduction in lifespan and gut homeostasis are not due to reduced sleep duration, but may derive from the loss of sleep quality as measured by arousal threshold.

**Fig 5:**
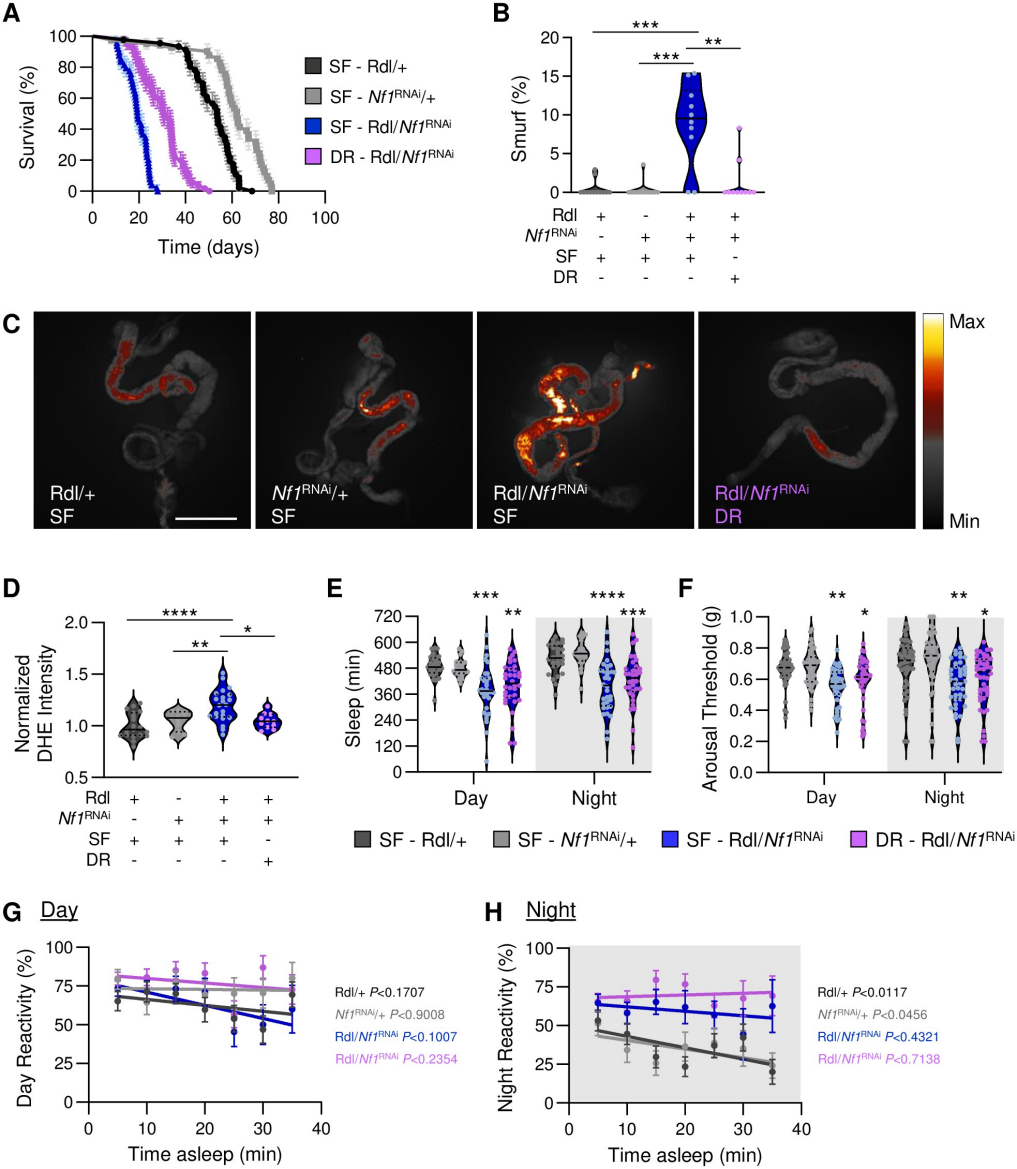
Dietary restriction in *Nf1*-deficient flies extends longevity and restores aging-associated phenotypes but has no effect on sleep depth. Flies were fed either a control, standard food (SF) diet or a dietary restricted (DR) diet. **A.** DR significantly extends longevity in flies with knockdown of *Nf1* in *Rdl*-expressing neurons (Log-Rank test: χ^2^ = 263.1, d.f. = 3, *P*<0.0001). N = 41–55. **B.** DR restores intestinal permeability in 20d flies with knockdown of *Nf1* in *Rdl*-expressing neurons (one-way ANOVA: F_3,40_ = 19.82, *P*<0.0001). N = 10–12. **(C,D)** ROS was measured in 20d flies by quantifying oxidized DHE. **C.** Oxidized DHE was measured in 20d control and *Nf1* knockdown flies fed either an SF or DR dietary regimen. Scale bar = 500μm. **D.** DR restores oxidized DHE signal intensity in 20d flies with knockdown of *Nf1* in *Rdl*-expressing neurons (one-way ANOVA: F_3,59_ = 9.332, *P*<0.0001). N = 11–22. **E.** There is a significant effect of genotype on sleep duration (two-way ANOVA: F_3,220_ = 26.46, *P*<0.0001). DR has no effect on sleep duration in flies with knockdown of *Nf1* in *Rdl*-expressing neurons during the day (*P*<0.6963) or the night (*P*<0.6145). N = 20–34. **(F-H)** Measurements of arousal threshold and reactivity using the DART system. **F.** There is a significant effect of genotype on arousal threshold (REML: F_3,339_ = 16.66, *P*<0.0001; N = 22–38). DR has no effect on arousal threshold in flies with knockdown of *Nf1* in *Rdl*-expressing neurons during the day (*P*<0.6594) or the night (*P*<0.7728). N = 41–46. **G.** Linear regression of daytime reactivity as a function of time asleep. The intercepts of each regression line are not significantly different from each other (F_3,990_ = 1.088, *P*<0.3532). **H.** Linear regression of nighttime reactivity as a function of time asleep. The intercepts of each regression line are significantly different from each other (F_3,994_ = 38.89, *P*<0.0001). N = 20–34. For sleep and gut measurements, the median (solid line) as well as 25th and 75th percentiles (dotted lines) are shown. For survival and reactivity measurements, error bars indicate ± SEM. The *P*-values in each panel indicate whether the slope of the regression line is significantly different from zero. White background indicates daytime, while gray background indicates nighttime. *p<0.05; **p<0.01; ***p<0.001; ****p<0.0001.

In flies, diet potently impacts both sleep and metabolic regulation [57–61], and long-term dietary restriction has been shown to extend lifespan [62–64]. Given the sleep, metabolic, and gut homeostasis phenotypes of *Nf1*-defficient flies, we sought to determine the impacts of dietary restriction on these diverse phenotypes. Flies were fed a standard food (SF) diet or a dietary restriction diet (DR), that differs only in the amount of yeast extract [65]. We first examined whether dietary restriction can restore the lifespan of flies with knockdown of *Nf1* in GABA_A_ receptor neurons. We found that in comparison to control food, DR significantly extends lifespan of *Nf1*-deficient flies, although it does not fully rescue to control levels (Fig 5A). We also measured the effects of DR on intestinal permeability and ROS. We found that DR rescued gut permeability and elevated ROS levels in Rdl-GAL4/*Nf1*^RNAi^ flies (Fig 5B–5D). This rescue of lifespan and aging traits by DR was achieved without any changes to sleep duration or sleep depth; dietary restricted Rdl-GAL4/*Nf1*^RNAi^ flies had significantly reduced arousal threshold and did not decreased reactivity during long nighttime sleep bouts (Fig 5E–5H), consistent with Rdl-GAL4/*Nf1*^RNAi^ flies on the SF diet. These findings suggest that dietary restriction is protective against the negative impacts of *Nf1* deficiency, despite having no effect on sleep duration or sleep quality.

## Discussion

Clinical evidence reveals Neurofibromin type 1 (NF1) to be critical for regulating diverse biological functions, as humans afflicted with neurofibromatosis type 1 have behavioral manifestations including a high co-morbidity with ADHD, autism, learning impairments, and sleep disruption [23,26,66–70]. Studies in mammalian models have revealed a robust role for *Nf1* in sleep and metabolic regulation, raising the possibility that they contribute to the complex systems in humans [71,72]. In *Nf1*-deficient *Drosophila*, metabolic rate is chronically elevated, sleep is shortened, and circadian rhythms are dysregulated in *Nf1* mutants [25,29,30,40,73–75], suggesting deep evolutionary conservation of *Nf1* function. The findings that *Nf1* plays a conserved role in regulating both sleep and metabolic rate raises the possibility that *Nf1* is a critical integrator of these processes and that it may play a role in various forms of metabolic dysfunction that are associated with sleep disturbance [76,77].

In *Drosophila* and mammals, sleep is associated with reduced metabolic rate [8,9,78]. In *Drosophila*, metabolic rate is elevated across the circadian cycle in *Nf1* mutants, and this is associated with reductions in energy stores and starvation resistance [40]. Here, we have applied indirect calorimetry to examine metabolic rate across individual sleep bouts and find that *Nf1* is required for reductions in metabolic rate associated with prolonged time spent asleep. These findings raise the possibility that Nf1 signaling is a sleep output that specifically serves to regulate metabolic rate. Supporting this notion, *Nf1* is proposed to be an output of the circadian clock because circadian gene expression is normal in *Nf1* mutants, yet flies are arrhythmic [25], and *Nf1* impacts the physiology of neurons downstream of clock circuits [75]. In *Drosophila*, numerous populations of neurons contribute to regulating sleep and wakefulness, presenting a challenge to localizing the integration of sleep and metabolic regulation [79]. The failure of *Nf1* mutant flies to integrate sleep and metabolic rate may provide a pathway to identify output from sleep neurons that regulate metabolic state.

There is growing evidence that *Drosophila*, like mammals, possess light and deep sleep [32]. For example, readouts of both broad electrical activity and defined neural circuits suggest light sleep associates with periods early in a sleep bout, while deeper sleep associates with periods later in a sleep bout [15,21,80]. Furthermore, periods later in a sleep bout are associated with an elevated arousal threshold that indicates deeper sleep [21]. These findings are supported by functional evidence that consolidation of sleep bouts is required for critical brain functions, including waste clearance and memory, and that these processes are impaired when sleep is disrupted [10,34,56]. Here, we provide evidence that *Nf1* flies fail to enter deep sleep, including sleep fragmentation, mathematical modeling of sleep pressure, reduced arousal threshold, and a loss of sleep-dependent reductions in metabolic rate. These findings suggest that *Nf1* mutants fail to enter deep sleep, even during prolonged sleep bouts. While it is possible that the lack of metabolic downscaling during sleep bouts is due to a lack of deep sleep, it is also possible that metabolic downscaling itself is required for deep sleep. Examining local field potentials and neural activity within *Nf1* mutants, as has been previously described [21,80], is likely to inform the neural basis for the loss of deep sleep.

*Nf1* is broadly expressed and regulates numerous behaviors and brain functions. For many behaviors, *Nf1* function has been localized to different subsets of neurons, suggesting localized changes in *Nf1* regulate distinct behaviors. For example, *Nf1* is broadly required within the pacemaker circuit to regulate 24-hour rhythms, while *Nf1* in the mushroom bodies regulates clock-dependent wakefulness [74,75]. For a number of other processes, including grooming behavior and metabolic rate, the specific population of neurons where *Nf1* functions has not been identified [29,40]. The broad and diverse effect of *Nf1* raises the possibility that it functions widely in many circuits, and that it may be challenging to localize its function to defined cell types. Here, we find that the metabolic and sleep phenotypes of *Nf1* mutant flies are phenocopied in flies with specific loss of *Nf1* in GABA_A_ receptor/*Rdl*-expressing neurons. These findings raise the possibility that sleep dysregulation is due to altered GABA signaling. Supporting this notion, GABA signaling to the mushroom bodies, the *Drosophila* memory center, is dysregulated in *Nf1* mutants [41]. It has also previously been reported that *Nf1* knockdown in GABA_A_ receptor neurons leads to shortened sleep bouts and reduced sleep duration [73]. These findings support the notion that Nf1 modulates GABA signaling. Future studies defining the specific population(s) of neurons where *Nf1* functions may reveal novel neural mechanisms regulating sleep-dependent regulation of metabolic rate.

Epidemiological data and individuals with chronic sleep loss reveal a link between shortened sleep duration and serious health problems [81,82]. Additionally, in several model organisms, sleep restriction can lead to premature death [48,83–86]. Lifespan is reduced in *Nf1* mutant flies [87], but its relationship to reduced sleep or circadian dysregulation has been unclear. In *Drosophila*, lifespan is reduced in short-sleeping genetic mutants and by chronic sleep deprivation [47–49]. However, there are many examples of short sleeping genetic mutants, as well as in artificially selected short-sleeping flies and chronically sleep deprived wildtype flies where lifespan is not impacted [50–52,88]. Therefore, further studies are required to more fully elucidate the interactions between sleep regulation and longevity in *Nf1* deficient flies, and it remains possible that sleep loss does not directly impact lifespan.

Sleep loss induced by acute manipulations in young flies, or during aging, results in increased sensitivity to ROS, suggesting the generation of ROS, or changes in clearing ROS, may be a critical function of sleep that is necessary for survival [89–91]. Further, evidence suggests that sleep deprivation leads to increased accumulation of ROS in the gut, resulting in gut permeability and death [49]. We find the ROS and permeability phenotypes in the guts of aged *Nf1* flies phenocopy those of animals that have been mechanically sleep deprived. Interestingly, chronic sleep deprivation did not change intestinal permeability, suggesting the decreased lifespan associated with chronic sleep loss is distinct from accelerated aging [49]. Evidence suggests ROS accumulation contributes to aging-related pathologies [92,93], including intestinal barrier dysfunction [53,94]. Our observations of both intestinal barrier dysfunction and ROS accumulation in the gut in aged *Nf1*-deficient flies suggests this genetic model may more faithfully phenocopy an accelerated aging genetic model. Together, these findings suggest that sleep consolidation, or deep sleep, is important for maintaining gut homeostasis. However, given the pleiotropic effects of *Nf1* on behavior and physiology, it remains a challenge to causally link this disruption of deep sleep with dysregulation of gut homeostasis. Future studies using other, less severe alleles of *Nf1*, in addition to other genetic models with reduced sleep quality will further elucidate this link. Ultimately, genetic models with reduced sleep quality may resemble human sleep disorders more closely than chronic sleep deprivation.

Dietary restriction (DR) extends lifespan from flies to humans [95–98], and has potent effects on gut health, including ROS and intestinal permeability [53,99–101]. In mammals, DR increases antioxidant defense during aging [102,103]. A previous study found that *Nf1* mutants have elevated ROS production and that this could be rescued by feeding antioxidants [87], supporting the notion that DR may be restorative in this genetic model. Our findings that DR can restore longevity and gut homeostasis, but not sleep depth in *Nf1*-deficient flies suggests sleep depth and gut homeostasis are separable. It is possible that the effects of *Nf1* on gut homeostasis are downstream of its effects on sleep, and that restoration of gut homeostasis with DR restores longevity without affecting sleep. Alternatively, *Nf1* may exert its effects on sleep depth and gut homeostasis through two different pathways, and defining the specific population(s) of neurons where *Nf1* functions may provide additional insight into this possibility. In mammals, DR reduces ROS production during aging, but is dependent on the duration of restriction [102]. Since our measurement of sleep depth was performed in young *Nf1*-deficient flies that have been maintained on a restricted diet for a short period of time, it is also possible that a longer duration of DR may ameliorate sleep depth in these flies. Future studies investigating how sleep depth changes during aging and whether it can also be rescued by DR will further our understanding of the interactions between diet and sleep regulation during aging.

Taken together, our findings reveal a novel and complex role for *Nf1* in regulating sleep depth. Loss of *Nf1* induces multiple phenotypes classically associated with a loss of deep sleep. Human mutations in *NF1* have been introduced to *Drosophila* and phenocopy many aspects of the human disease [40,104–106]. Future studies examining the effects of human *NF1* alleles, as well as additional *Drosophila* alleles that result in similar interactions between sleep depth, longevity, and gut homeostasis, will help to elucidate whether these phenotypes can be dissociated. In addition, the identification of these phenotypes in *Nf1* mutants allow for future work assessing the contributions of antioxidants, additional sleep promoting drugs, and sleep-regulating environmental stimuli on longevity and gut function [49,107]. Therefore, these findings establish *Nf1* mutants as a model to study the function of deep sleep and provide the ability to investigate the function of disease-causing mutations on sleep regulation.

## Methods

### Fly husbandry and stocks

Flies were grown and maintained on standard *Drosophila* food media (Bloomington Recipe, Genesee Scientific, San Diego, California) in incubators (Powers Scientific, Warminster, Pennsylvania) at 25°C on a 12:12 LD cycle with humidity set to 55–65%. The following fly strains were obtained from the Bloomington Stock Center: *w*^1118^ (#5905); Nsyb-GAL4 (#39171; [108]; *Rdl*-GAL4 (#66509); *Rdl*-GAL4 (#84688; [109]UAS-mcd8::GFP (#32186; [110]; and UAS-*Nf1*^RNAi2^ (#25845; [111] The *nf1*^P1^ and *Nf1*^RNAi^ (UAS-*Nf1*^RNAi^;UAS-dicer2) lines were used previously [40,112]. All lines were backcrossed to the *w*^1118^ laboratory strain for 10 generations. Unless otherwise stated, 3-to-5 day old mated males were used for all experiments performed in this study. For experiments using aged flies, flies were maintained on standard food and transferred to fresh vials every other day.

### Sleep and activity

For experiments using the *Drosophila* Activity Monitoring (DAM) system (Trikinetics, Waltham, MA, USA), measurements of sleep and waking activity were measured as previously described [17,18]. For each individual fly, the DAM system measures activity by counting the number of infrared beam crossings over time. These activity data were then used to calculate sleep, defined as bouts of immobility of 5 min or more. Sleep traits were then extracted using the *Drosophila* Sleep Counting Macro [113].

### Arousal threshold and reactivity

Arousal threshold was measured using the *Drosophila* Arousal Tracking system (DART), as previously described [32]. In brief, individual male flies were loaded into plastic tubes (Trikinectics, Waltham, Massachusetts) and placed onto trays containing vibrating motors. Flies were recorded continuously using a USB-webcam (QuickCam Pro 900, Logitech, Lausanne, Switzerland) with a resolution of 960x720 at 5 frames per second. The vibrational stimulus, video tracking parameters, and data analysis were performed using the DART interface developed in MATLAB (MathWorks, Natick, Massachusetts). To track fly movement, raw video flies were subsampled to 1 frame per second. Fly movement, or a difference in pixelation from one frame to the next, was detected by subtracting a background image from the current frame. The background image was generated as the average of 20 randomly selected frames from a given video. Fly activity was measured as movement of greater than 3 mm. Sleep was determined by the absolute location of each fly and was measured as bouts of immobility for 5 min or more. Arousal threshold was assessed using sequentially increasing vibration intensities, from 0 to 1.2 g, in 0.3 g increments, with an inter-stimulus delay of 15 s, once per hour over 24 hours starting at ZT0. Measurements of arousal threshold are reported as the proportion of the maximum force applied to the platform, thus an arousal threshold of 0.4 is 40% of 1.2g. Reactivity was assessed at the maximum stimulus intensity of 1.2 g, with an inter-stimulus delay of 15 s, once per hour over 24 hours starting at ZT0. Measurements of reactivity are reported as the percentage of sleeping flies that are responsive to the stimulus.

### Indirect calorimetry

Metabolic rate was measured using the Sleep and Activity Metabolic Monitor (SAMM) system, as previously described [8,9]. Briefly, male flies were placed individually into behavioral chambers containing a food vial of 1% agar and 5% sucrose. Flies were acclimated to the chambers for 24 hrs and then metabolic rate was assessed by quantifying the amount of CO_2_ produced in 5 min intervals during the subsequent 24hrs. To investigate how CO_2_ production may change with time spent asleep, sleep and activity were measured simultaneously using the *Drosophila* Locomotor Activity Monitor System. The percent change in VCO_2_ over the duration of a single sleep bout was calculated using the following equation: [(VCO_2_ @ 5 min)–(VCO_2_ @ 10 min)] / (VCO_2_ @ 5min) × 100. This was repeated for each 5 min bin of sleep, for the entire length of the sleep bout. Since a single fly typically has multiple sleep bouts, the percent change in VCO_2_ for each 5 min bin of sleep was averaged across all sleep bouts over the course of the day/night.

### Immunohistochemistry

For brain dissections, brains of three to five day old male flies were dissected in ice-cold phosphate buffered saline (PBS) and fixed in 4% formaldehyde, PBS, and 0.5% Triton-X for 35 min at room temperature, as previously described [114]. Brains were then rinsed 3x with cold PBS and 0.5% Triton-X (PBST) for 10 min at room temperature and then overnight at 4°C. The following day, the brains were incubated for 24 hours in primary antibody (1:20 mouse nc82; Iowa Hybridoma Bank; The Developmental Studies Hybridoma Bank, Iowa City, Iowa), and then diluted in 0.5% PBST at 4°C on a rotator. The following day, the brains were rinsed 3x in cold PBST for 10 min at room temperature and then incubated in secondary antibody (1:400 donkey anti-rabbit Alexa 488 and 1:400 donkey anti-mouse Alexa 647; ThermoFisher Scientific, Waltham, Massachusetts) for 95 min at room temperature. The brains were again rinsed 3x in cold PBST for 10 min at room temperature, then stored overnight in 0.5% PBST at 4°C. Lastly, the brains were mounted in Vectashield Antifade Mounting Medium (H-1000; VECTOR Laboratories, Burlingame, California) between a glass slide and coverslip, and then imaged in 1μm sections on a Nikon A1R confocal microscope (Nikon, Tokyo, Japan) using a 20X oil immersion objective. For gut dissections, guts were dissected using similar protocols. Briefly, guts were dissected and then fixed for 5 min. Guts were then rinsed 3x in cold PBST for 5 min and mounted in Vectashield with DAPI (H-1200). Guts were then imaged in 2.7 μm sections using a 10X air objective. Images are presented as the Z-stack projection through the entire brain and processed using ImageJ2.

### Longevity

Longevity was measured using the DAM system. Freshly emerged flies were isolated and provided time to mate for 2 days. Male flies were then separated by anesthetizing with mild CO_2_ and loaded into tubes containing standard food. Flies were flipped to new tubes containing fresh standard food every 5 days. The time of death was manually determined for each individual fly as the last bout of waking activity. The lifespan of a fly was calculated as the number of days it survived post-emergence.

### Intestinal permeability

Intestinal integrity was assessed using the Smurf assay, as previously described [53,54]. First, freshly emerged flies were isolated and provided time to mate for 2 days. Male flies were then separated by anesthetizing with mild CO_2_ and placed into vials containing standard food at a density of ~20 flies per vial. At ZT 0, flies of a given age and genotype were transferred onto fresh medium containing blue dye (2.5% w/v; FD&C blue dye #1) for 24 hrs. At ZT 0 the following day, the percentage of Smurf flies in each vial was recorded. Flies were considered Smurf if blue coloration extended beyond the gut.

### ROS imaging and quantification

*In situ* ROS detection was performed using dihydroethidium (DHE; D11347, ThermoFisher Scientific), as previously described [49,115]. At ZT 0–2, flies were anesthetized on ice and whole guts were dissected in Gibco Schneider’s *Drosophila* Medium (21720024, ThermoFisher Scientific). The tissue was then incubated at room temperature with 60 μm DHE for 5 min in the dark. Next, tissues were washed 3x in Schneider’s medium for 5 min and then once in PBS for 5 min. Samples were then mounted in Vectashield with DAPI between a glass slide and coverslip and then imaged immediately on a Nikon A1R confocal microscope (Nikon) using a 10X air objective. Total ROS levels were quantified from pixel intensities of the Z-stack projection (sum slices). An ROI (gut tissue) was determined from the DAPI channel and then the mean of the summed DHE intensity averaged from each tissue was used for statistical analysis. Images are presented as the Z-stack projection through the entire gut and were processed using ImageJ2.

### Dietary and pharmacological manipulations

For sleep induction experiments, gaboxadol was added to melted standard *Drosophila* media at a concentration of 0.1 mg/mL (T101, Sigma), as used previously to promote sleep in flies [34,55,56]. For antioxidant feeding experiments, antioxidants were diluted in EtOH and then individually added to standard *Drosophila* media. The antioxidants used were previously described to rescue accumulation of ROS in the gut due to chronic sleep deprivation at the following concentrations: 100ug/mL melatonin and 2mM Lipoic acid [49]. Control flies received the EtOH solvent alone. For dietary restriction (DR) experiments, DR was induced by feeding flies a protein-restricted diet, as previously described [65]. Briefly, control flies were placed on the Caltech high-protein, standard food diet (SF) diet (8.6% cornmeal 5% sucrose, 0.46% agar, 1% acid mix, and 5% yeast extract), whereas experimental flies were fed a DR diet that differs only in the amount of yeast extract (0.5% yeast extract). The Caltech standard food diet is different from the Bloomington standard food diet used in the experiments described above, which differ in their protein:carbohydrate ratio [116]. All experiments were carried out similarly as described above. For experiments using aged flies, flies were transferred to fresh vials every other day.

### Statistical analysis

Measurements of sleep duration, metabolic rate, and DHE intensity are presented as bar graphs displaying the mean ± standard error. Unless otherwise noted, a one-way or two-way analysis of variance (ANOVA) was used for comparisons between two or more genotypes and one treatment or two or more genotypes and two treatments, respectively. Measurements of arousal threshold and intestinal permeability were not normally distributed and so are presented as violin plots; indicating the median, 25^th^, and 75^th^ percentiles. The non-parametric Kruskal-Wallis test was used to compare two or more genotypes. To compare two or more genotypes and two treatments, a restricted maximum likelihood (REML) estimation was used. Linear regression analyses were used to characterize the relationship between the change in CO_2_ output and time spent asleep as well as between reactivity and time spent asleep. An F-test was used to determine whether the slope of each regression line was different from zero, while an ANCOVA was used to compare the slopes of different treatments. To assess differences in survivorship, longevity was analyzed using a log-rank test. All *post hoc* analyses were performed using Sidak’s multiple comparisons test. A minimum of two independent runs were performed for each experiment, while the sample size for each genotype/treatment are presented in the Fig legends. All statistical analyses were performed using InStat software (GraphPad Software 8.0).

## Supporting information

S1 Fig Loss of *Nf1* increases waking activity. Waking activity was measured as the number of beam crosses per waking minute. **A**. There is a significant effect of genotype on waking activity (two-way ANOVA: F_2,172_ = 42.73, *P*<0.0001). Compared to control and heterozygote flies, *nf1*^P1^ mutants are significantly more active during the day (+, *P*<0.0001; het, *P*<0.0001), but not night (+, *P*<0.1689; het, *P*<0.2407). N = 26–32. **B**. There is a significant effect of genotype on waking activity (two-way ANOVA: F_2,314_ = 16.60, *P*<0.0001). Compared to controls, pan-neuronal knockdown of *Nf1* significantly increases waking activity during the day (nsyb/+, *P*<0.0389; *Nf1*^RNAi^/+, *P*<0.0001) and night (nsyb/+, *P*<0.0049; *Nf1*^RNAi^/+, *P*<0.0036). N = 50–55. The median (solid line) as well as 25th and 75th percentiles (dotted lines) are shown. *p<0.05; ****p<0.0001.
(TIF)

S2 Fig Pan-neuronal knockdown of *Nf1* significantly reduces sleep duration and sleep depth using an independent RNAi line. **(A-F).** Sleep and activity traits of pan-neuronal *Nf1*^RNAi2^ knockdown flies and their respective controls. **A**. There is a significant effect of genotype on sleep duration (two-way ANOVA: F_2,378_ = 79.47, *P*<0.0001). Compared to controls, pan-neuronal knockdown of *Nf1* significantly reduces sleep during the day (nsyb/+, *P*<0.0001; *Nf1*^RNAi2^/+, *P*<0.0001) and night (nsyb/+, *P*<0.0001; *Nf1*^RNAi2^/+, *P*<0.0001). **B**. There is a significant effect of genotype on bout number (two-way ANOVA: F_2,378_ = 2.679, *P*<0.0499). Compared to controls, pan-neuronal knockdown of *Nf1* significantly increases bout number during the night (nsyb/+, *P*<0.0195; *Nf1*^RNAi2^/+, *P*<0.0373), but not during the day (nsyb/+, *P*<0.8369; *Nf1*^RNAi2^/+, *P*<0.6346). **C**. There is a significant effect of genotype on bout length (two-way ANOVA: F_2,378_ = 18.02, *P*<0.0001). Compared to controls, pan-neuronal knockdown of *Nf1* significantly reduces bout length during the ngiht (nsyb/+, *P*<0.0001; *Nf1*^RNAi2^/+, *P*<0.0001), but not during the day (nsyb/+, *P*<0.5054; *Nf1*^RNAi2^/+, *P*<0.6506). **D**. There is a significant effect of genotype on waking activity (two-way ANOVA: F_2,378_ = 17.32, *P*<0.0001). Compared to controls, pan-neuronal knockdown of *Nf1* significantly increases waking activity during the day (nsyb/+, *P*<0.0035; *Nf1*^RNAi^/+, *P*<0.0001), but not during the day (nsyb/+, *P*<0.1695; *Nf1*^RNAi^/+, *P*<0.1646). **E**. There is a significant effect of genotype on the probability of falling asleep (two-way ANOVA: F_2,378_ = 71.99, *P*<0.0001). P(Doze) is significantly lower upon knockdown of *Nf1* during the day (nsyb/+, *P*<0.0071; *Nf1*^RNAi^/+, *P*<0.0001) and night (nsyb/+, *P*<0.0001; *Nf1*^RNAi^/+, *P*<0.0001). **F**. There is a significant effect of genotype on the probability of waking up (two-way ANOVA: F_2,378_ = 41.99, *P*<0.0001). P(Wake) is significantly higher upon knockdown of *Nf1* during the day (nsyb/+, *P*<0.0072; *Nf1*^RNAi^/+, *P*<0.0004) and night (nsyb/+, *P*<0.0001; *Nf1*^RNAi^/+, *P*<0.0001). N = 60–66. **(G,H)** Linear regression of **(G)** daytime and **(H)** nighttime reactivity as a function of time asleep in *Nf1*^RNAi^ knockdown flies and their controls. During the day, the slopes of each regression line are not significantly different from each other (F_2,816_ = 1.243, *P* = 0.2890). During the night, the slopes of each regression line are significantly different from each other (F_2,823_ = 3.504, *P* = 0.0305). N = 29–40. For violin plots, the median (solid line) as well as 25th and 75th percentiles (dotted lines) are shown. For reactivity measurements, error bars indicate ± SEM. The *P*-values in each panel indicate whether the slope of the regression line is significantly different from zero. White background indicates daytime, while gray background indicates nighttime. **p<0.01; ***p<0.001; ****p<0.0001.
(TIF)

S3 Fig Probabilistic analysis suggests that loss of *Nf1* increases the probability of waking. **(A-D)** Computational modeling of waking probabilities. **A**. Profiles of the probability of waking up in *nf1*^P1^ mutants, heterozygotes, and their control. **B**. There is a significant effect of genotype on the probability of waking up (two-way ANOVA: F_2,172_ = 144.6, *P*<0.0001). P(Wake) is significantly higher in *nf1*^P1^ mutant flies during the day (+, *P*<0.0001; het, *P*<0.0001) and night (+, *P*<0.0001; het, *P*<0.0001). **C**. Profiles of the probability of waking up in pan-neuronal *Nf1*^RNAi^ knockdown flies and their controls. **D**. There is a significant effect of genotype on the probability of waking up (two-way ANOVA: F_2,314_ = 67.34, *P*<0.0001). P(Wake) is significantly higher upon knockdown of *Nf1* during the day (nsyb/+, *P*<0.0001; *nf1*^RNAi^/+, *P*<0.0001) and night (nsyb/+, *P*<0.0001; *Nf1*^RNAi^/+, *P*<0.0001). N = 26–32. **(E-H)** Computational modeling of sleep probabilities. **E**. Profiles of the probability of falling asleep in *nf1*^P1^ mutants, heterozygotes, and their control. **F**. There is a significant effect of genotype on the probability of falling asleep (two-way ANOVA: F_2,172_ = 23.10, *P*<0.0001). P(Doze) is significantly lower in *nf1*^P1^ mutant flies during the day (+, *P*<0.0001; het, *P*<0.0001), but there is no difference during the night (+, *P*<0.0001; het, *P*<0.0001). **G**. Profiles of the probability of falling asleep in pan-neuronal *Nf1*^RNAi^ knockdown flies and their controls. **H**. There is a significant effect of genotype on the probability of falling asleep (two-way ANOVA: F_2,314_ = 13.55, *P*<0.0001). P(Doze) is significantly lower upon knockdown of *Nf1* during the day (nsyb/+, *P*<0.0001; *Nf1*^RNAi^/+, *P*<0.0001), but there is no difference during the night (nsyb/+, *P*<0.8053; *Nf1*^RNAi^/+, *P*<0.9999). N = 50–55. For profiles, shaded regions indicate ± SEM. White background indicates daytime, while gray background indicates nighttime. ZT indicates zeitgeber time. For violin plots, the median (solid line) as well as 25th and 75th percentiles (dotted lines) are shown. ****p<0.0001.
(TIF)

S4 Fig Loss of *Nf1* decreases sleep in the DART system. **A**. There is a significant effect of genotype on sleep duration (two-way ANOVA: F_2,372_ = 131.7, *P*<0.0001). Compared to control and heterozygote flies, *nf1*^P1^ mutants sleep significantly less during the day (+, *P*<0.0001; het, *P*<0.0001) and night (+, *P*<0.0001; het, *P*<0.0001). N = 58–70. **B**. There is a significant effect of genotype on sleep duration (two-way ANOVA: F_2,220_ = 19.05, *P*<0.0001). Compared to controls, pan-neuronal knockdown of *Nf1* significantly reduces sleep during the day (nsyb/+, *P*<0.0003; *Nf1*^RNAi^/+, *P*<0.0001) and night (nsyb/+, *P*<0.0106; *Nf1*^RNAi^/+, *P*<0.0135). N = 36–41. The median (solid line) as well as 25th and 75th percentiles (dotted lines) are shown. *p<0.05; ***p<0.001; ****p<0.0001.
(TIF)

S5 Fig Loss of *Nf1* decreases sleep and increases metabolic rate in the SAMM system. Sleep duration and metabolic rate were measured in the SAMM system. **A**. There is a significant effect of genotype on sleep duration (two-way ANOVA: F_2,154_ = 10.92, *P*<0.0001). Compared to control and heterozygote flies, *nf1*^P1^ mutants sleep significantly less during the day (+, *P*<0.0414; het, *P*<0.0159) and night (+, *P*<0.0167; het, *P*<0.0033). **B**. There is a significant effect of genotype on metabolic rate (two-way ANOVA: F_2,154_ = 43.72, *P*<0.0001). Compared to control and heterozygote flies, *nf1*^P1^ mutants significantly increase CO_2_ output during the day (+, *P*<0.0001; het, *P*<0.0001) and night (+, *P*<0.0001; het, *P*<0.0001). N = 26–27. **C**. There is a significant effect of genotype on sleep duration (two-way ANOVA: F_2,224_ = 13.79, *P*<0.0001). Compared to controls, pan-neuronal knockdown of *Nf1* significantly decreases sleep during the day (nsyb/+, *P*<0.0001; *Nf1*^RNAi^/+, *P*<0.0001), but not the night (nsyb/+, *P*<0.3333; *Nf1*^RNAi^/+, *P*<0.2203). **D**. There is a significant effect of genotype on metabolic rate (two-way ANOVA: F_2,224_ = 136.0, *P*<0.0001). Compared to controls, pan-neuronal knockdown of *Nf1* significantly increases CO_2_ output during the day (nsyb/+, *P*<0.0001; *Nf1*^RNAi^/+, *P*<0.0001) and night (nsyb/+, *P*<0.0001; *Nf1*^RNAi^/+, *P*<0.0001). N = 30–44. The median (solid line) as well as 25th and 75th percentiles (dotted lines) are shown. *p<0.05; **p<0.01; ****p<0.0001.
(TIF)

S6 Fig Loss of *Nf1* increases metabolic rate during waking and sleeping. **A**. There is a significant effect of genotype on metabolic rate during waking (two-way ANOVA: F_2,154_ = 16.76, *P*<0.0001). In the daytime, *nf1*^P1^ mutants significantly increase waking CO_2_ output compared to control and heterozygote flies (+, *P*<0.0003; het, *P*<0.0412). At night, *nf1*^P1^ mutants significantly increase waking CO_2_ output compared to control flies (+, *P*<0.0002), with heterozygotes being intermediate (het, *P*<0.0762). **B**. There is a significant effect of genotype on metabolic rate during sleep (two-way ANOVA: F_2,154_ = 53.48, *P*<0.0001). Compared to control and heterozygote flies, *nf1*^P1^ mutants significantly increase CO_2_ output during sleep during the day (+, *P*<0.0001; het, *P*<0.0001) and night (+, *P*<0.0001; het, *P*<0.0001). N = 26–27. **C**. There is a significant effect of genotype on metabolic rate during waking (two-way ANOVA: F_2,224_ = 97.10, *P*<0.0001). Compared to controls, pan-neuronal knockdown of *Nf1* significantly increases waking CO_2_ output during the day (nsyb/+, *P*<0.0001; *Nf1*^RNAi^/+, *P*<0.0001) and night (nsyb/+, *P*<0.0001; *Nf1*^RNAi^/+, *P*<0.0001). **D**. There is a significant effect of genotype on metabolic rate during sleep (two-way ANOVA: F_2,224_ = 176.1, *P*<0.0001). Compared to controls, pan-neuronal knockdown of *Nf1* significantly increases CO_2_ output during sleep during the day (nsyb/+, *P*<0.0001; *Nf1*^RNAi^/+, *P*<0.0001) and night (nsyb/+, *P*<0.0001; *Nf1*^RNAi^/+, *P*<0.0001). N = 30–44. The median (solid line) as well as 25th and 75th percentiles (dotted lines) are shown. *p<0.05; ***p<0.001; ****p<0.0001.
(TIF)

S7 Fig Computational modeling of sleep and waking probabilities upon knockdown of *Nf1* in *Rdl*-expressing neurons. **A**. There is a significant effect of genotype on the probability of falling asleep (two-way ANOVA: F_2,362_ = 27.27, *P*<0.0001). Compared to controls, knockdown of *Nf1* in *Rdl*-expressing neurons significantly reduces P(Dose) during the day (*Rdl*/+, *P*<0.0097; *Nf1*^RNAi^/+, *P*<0.0372), but only compared to one control during the night (*Rdl*/+, *P*<0.1412; *Nf1*^RNAi^/+, *P*<0.0001). **B**. There is a significant effect of genotype on the probability of waking up (two-way ANOVA: F_2,362_ = 161.5, *P*<0.0001). Compared to controls, knockdown of *Nf1* in *Rdl*-expressing neurons significantly increases P(Wake) and occurs during the day (*Rdl*/+, *P*<0.0001; *Nf1*^RNAi^/+, *P*<0.0001) and night (*Rdl*/+, *P*<0.0001; *Nf1*^RNAi^/+, *P*<0.0001). N = 57–66. The median (solid line) as well as 25th and 75th percentiles (dotted lines) are shown. *p<0.05; ****p<0.0001.
(TIF)

S8 Fig Knockdown of *Nf1* in *Rdl*-expressing neurons decreases sleep and increases metabolic rate. **A**. There is a significant effect of genotype on sleep duration in the DART system (two-way ANOVA: F_2,378_ = 27.38, *P*<0.0001). Compared to controls, knockdown of *Nf1* in *Rdl*-expressing neurons significantly reduces sleep and occurs during day (*Rdl*/+, *P*<0.0012; *Nf1*^RNAi^/+, *P*<0.0001) and night (*Rdl*/+, *P*<0.0015; *Nf1*^RNAi^/+, *P*<0.0001). N = 49–61. **B**. There is a significant effect of genotype on sleep duration in the SAMM system (two-way ANOVA: F_2,188_ = 4.708, *P*<0.0101). Compared to controls, knockdown of *Nf1* in *Rdl*-expressing neurons significantly reduces sleep, but only occurs during the day (*Rdl*/+, *P*<0.0001; *Nf1*^RNAi^/+, *P*<0.0001) and not the night (*Rdl*/+, *P*<0.9410; *Nf1*^RNAi^/+, *P*<0.2371). **C**. There is a significant effect of genotype on metabolic rate during waking (two-way ANOVA: F_2,188_ = 54.14, *P*<0.0001). Compared to controls, knockdown of *Nf1* in *Rdl*-expressing neurons significantly increases CO_2_ output during the day (*Rdl*/+, *P*<0.0001; *Nf1*^RNAi^/+, *P*<0.0001) and night (*Rdl*/+, *P*<0.0003; *Nf1*^RNAi^/+, *P*<0.0001). **D**. There is a significant effect of genotype on metabolic rate during sleep (two-way ANOVA: F_2,188_ = 136.1, *P*<0.0001). Compared to controls, knockdown of *Nf1* in *Rdl*-expressing neurons significantly increases CO_2_ output during the day (*Rdl*/+, *P*<0.0001; *Nf1*^RNAi^/+, *P*<0.0001) and night (*Rdl*/+, *P*<0.0001; *Nf1*^RNAi^/+, *P*<0.0001). N = 30–35. The median (solid line) as well as 25th and 75th percentiles (dotted lines) are shown. **p<0.01; ****p<0.0001.
(TIF)

S9 Fig Selective knockdown of *Nf1* in GABA_A_ receptor neurons in the brain significantly increases reactivity. The *tsh*-GAL80 repressor was used to restrict expression of *Nf1*^RNAi^ to GABA_A_ receptor neurons in the brain. (A,B). The expression pattern of *tsh*-GAL80; *Rdl*-GAL4 neurons is visualized with GFP in the brain (A) and the gut (B). For the brain, background staining is NC82 antibody (magenta). Scale bar = 100μm. For the gut, background staining is DAPI (blue). Scale bar = 1000μm. (C,D) Measurements of reactivity in *Nf1*^RNAi^ knockdown flies and their respective controls using the DART system. C. Linear regression of daytime reactivity as a function of time asleep in *Nf1*^RNAi^ knockdown flies and their controls. The intercepts of each regression line are significantly different from each other (F_2,1416_ = 71.71, *P*<0.0001). D. Linear regression of nighttime reactivity as a function of time asleep in *Nf1*^RNAi^ knockdown flies and their controls. The intercepts of each regression line are significantly different from each other (F_2,1724_ = 76.56, *P*<0.0001). N = 52–71. Error bars indicate ± SEM. The *P*-values in each panel indicate whether the slope of the regression line is significantly different from zero. White background indicates daytime, while gray background indicates nighttime.
(TIF)

S10 Fig Knockdown of *Nf1* has no effect on fluorescence intensity of GABA_A_ receptor neurons. GABA_A_ receptor neurons were targeted using the *Rdl*-GAL4 driver. **(A,B)**. The expression pattern of *Rdl*-expressing neurons is visualized with GFP. Background staining is NC82 antibody (magenta). Scale bar = 100μm. **C**. Knockdown of *Nf1* in *Rdl*-expressing neurons has no effect on fluorescence intensity (t-test: t_25_ = 0.7508, *P*<0.4598). The median (solid line) as well as 25th and 75th percentiles (dotted lines) are shown.
(TIF)

S11 Fig Loss of *Nf1* promotes aging-associated phenotypes. **A**. Compared to control flies, loss of *Nf1* significantly decreases longevity (Log-Rank test: χ^2^ = 209.0, d.f. = 2, *P*<0.0001). N = 70–84. **B**. Compared to controls, pan-neuronal knockdown of *Nf1* significantly decreases longevity (Log-Rank test: χ^2^ = 253.4, d.f. = 2, *P*<0.0001). N = 80–96. **C.** There is a significant effect of genotype on intestinal permeability (two-way ANOVA: F_1,42_ = 29.45, *P*<0.0002). Loss of *Nf1* does not change intestinal barrier dysfunction in 5d flies (+, *P*<0.0565; het, *P*<0.0648), but significantly increases in 20d flies (+, *P*<0.0001; het, *P*<0.0001). N = 9–13. **D.** There is a significant effect of genotype on intestinal permeability (two-way ANOVA: F_2,65_ = 18.80, *P*<0.0001). Pan-neuronal knockdown of *Nf1* does not change intestinal barrier dysfunction in 5d flies (nsyb/+, *P*<0.2093; *Nf1*^RNAi^/+, *P*<0.1973), but significantly increases in 20d flies (nsyb/+, *P*<0.0001; *Nf1*^RNAi^/+, *P*<0.0001). N = 9–12. The median (solid line) as well as 25th and 75th percentiles (dotted lines) are shown. ***p<0.001; ****p<0.0001.
(TIF)

S12 Fig ROS in the gut increases upon pan-neuronal knockdown of *Nf1*. ROS was measured in 5d and 20d flies by quantifying oxidized DHE levels. **A.** Oxidized DHE was measured in 5d control and *Nf1* knockdown flies. **B**. Pan-neuronal knockdown of *Nf1* significantly increases oxidized DHE signal intensity in 5d flies (one-way ANOVA: F_2,51_ = 30.37, *P*<0.0001). N = 13–21. **C**. Oxidized DHE was measured in 20d control and *Nf1* knockdown flies. **D**. Pan-neuronal knockdown of *Nf1* significantly increases oxidized DHE signal intensity in 20d flies (one-way ANOVA: F_2,47_ = 16.36, *P*<0.0001). N = 10–28. Scale bar = 500μm. The median (solid line) as well as 25th and 75th percentiles (dotted lines) are shown. ***p<0.001; ****p<0.0001.
(TIF)

S13 Fig Antioxidant feeding has no effect on lifespan or gut homeostasis in *Nf1*-deficient flies. The antioxidants Lipoic Acid (LiA) or Melatonin (Mel) were added to standard food. A. Antioxidant feeding has no effect on longevity in Rdl-GAL4/*Nf1*^RNAi^ flies (Log-Rank test: χ^2^ = 1.665, d.f. = 2, *P*<0.4351). N = 46–48. B. There is significant effect of genotype on intestinal permeability in 20d flies upon knockdown of *Nf1* in *Rdl*-expressing neurons (one-way ANOVA: F_4,37_ = 7.634, *P*<0.0001), but no effect of antioxidant feeding among *Nf1*-deficient flies (one-way ANOVA: F_2,19_ = 0.7633, *P*<0.4799). N = 11–22. C. Oxidized DHE was measured in 20d flies fed either standard food or standard food with antioxidants. Scale bar = 500μm. D. There is significant effect of genotype on DHE signal intensity in 20d flies upon knockdown of *Nf1* in *Rdl*-expressing neurons (one-way ANOVA: F_4,82_ = 18.02, *P*<0.0001), but no effect of antioxidant feeding among *Nf1*-deficient flies (one-way ANOVA: F_2,61_ = 2.2682, *P*<0.1122). N = 11–22. For survival measurements, error bars indicate ± SEM. For gut measurements, the median (solid line) as well as 25th and 75th percentiles (dotted lines) are shown. *p<0.05; ****p<0.0001.
(TIF)

S14 Fig Gaboxadol feeding promotes sleep in *Nf1*-deficient flies, but has no effect on sleep depth, lifespan, or gut homeostasis. Gaboxadol (*Gabox*; 0.1 mg/mL) was added to the fly diet to promote sleep. **A.** There is a significant effect of gaboxadol on daytime sleep duration (two-way ANOVA: F_1,182_ = 59.14, *P*<0.0001). Gaboxadol increases daytime sleep in all genotypes tested (*Rdl*/+, *P*<0.0023; *Nf1*^RNAi^/+, *P*<0.0001; *Rdl*/*Nf1*^RNAi^, *P*<0.0001). **B.** There is a significant effect of gaboxadol on nighttime sleep duration (two-way ANOVA: F_1,182_ = 92.63, *P*<0.0001). Gaboxadol increases nighttime sleep in all genotypes tested (*Rdl*/+, *P*<0.0001; *Nf1*^RNAi^/+, *P*<0.0018; *Rdl*/*Nf1*^RNAi^, *P*<0.0001). N = 30–32. **C.** There is a significant effect of gaboxadol on daytime arousal threshold (REML: F_1,155_ = 15.41, *P*<0.0001). Gaboxadol increases daytime arousal threshold in control flies, but not in flies with knockdown of *Nf1* in *Rdl*-expressing neurons (*Rdl*/+, *P*<0.0080; *Nf1*^RNAi^/+, *P*<0.0001; *Rdl*/*Nf1*^RNAi^, *P*<0.5755). **D.** There is a significant effect of gaboxadol on nighttime arousal threshold (REML: F_1,132_ = 30.44, *P*<0.0001). Gaboxadol increases nighttime arousal threshold in control flies, but not in flies with knockdown of *Nf1* in *Rdl*-expressing neurons (*Rdl*/+, *P*<0.0001; *Nf1*^RNAi^/+, *P*<0.0029; *Rdl*/*Nf1*^RNAi^, *P*<0.4634). N = 20–34. **E.** Gaboxadol significantly extends longevity in control Rdl-GAL4/+ flies (Log-Rank test: χ^2^ = 15.36, d.f. = 1, *P*<0.0001). N = 34–37. **F.** Gaboxadol significantly extends longevity in control *Nf1*^RNAi^/+ flies (Log-Rank test: χ^2^ = 15.51, d.f. = 1, *P*<0.0001). N = 37–40. **G.** Gaboxadol has no effect on longevity in Rdl-GAL4/ *Nf1*^RNAi^ flies (Log-Rank test: χ^2^ = 1.904, d.f. = 1, *P*<0.1677). N = 43–44. **H.** Gaboxadol has no effect on intestinal permeability in 20d flies with knockdown of *Nf1* in *Rdl*-expressing neurons (one-way ANOVA: F_3,35_ = 9.357, *P*<0.0001). N = 9–10. **(I,J)** ROS was measured in 20d flies by quantifying oxidized DHE. **I.** Oxidized DHE was measured in 20d flies fed either standard food or standard food with Gaboxadol. Scale bar = 500μm. **J.** Gaboxadol has no effect on oxidized DHE signal intensity in 20d flies with knockdown of *Nf1* in *Rdl*-expressing neurons (one-way ANOVA: F_3,87_ = 13.01, *P*<0.0001). N = 19–27. For sleep and gut measurements, the median (solid line) as well as 25th and 75th percentiles (dotted lines) are shown. For survival measurements, error bars indicate ± SEM. White background indicates daytime, while gray background indicates nighttime. **p<0.01; ***p<0.001; ****p<0.0001.
(TIF)

S1 Data Supplementary Data. (XLSX)
