# Supplementary figures and images for: Neurofibromin 1 mediates sleep depth in Drosophila
Source: PLoS Genet. 2023 Dec 13;19(12):e1011049. doi: 10.1371/journal.pgen.1011049 (PMC10763969; doi:10.1371/journal.pgen.1011049)

Supplemental Figure 1

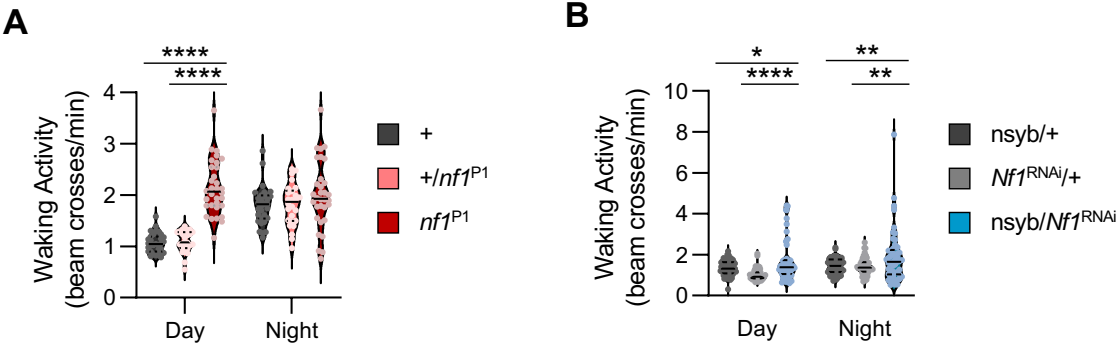

Supplement: S1 Fig — Waking activity was measured as the number of beam crosses per waking minute. A. There is a significant effect of genotype on waking activity (two-way ANOVA: F2,172 = 42.73, P<0.0001). Compared to control and heterozygote flies, nf1P1 mutants are significantly more active during the day (+, P<0.0001; het, P<0.0001), but not night (+, P<0.1689; het, P<0.2407). N = 26–32. B. There is a significant effect of genotype on waking activity (two-way ANOVA: F2,314 = 16.60, P<0.0001). Compared to controls, pan-neuronal knockdown of Nf1 significantly increases waking activity during the day (nsyb/+, P<0.0389; Nf1RNAi/+, P<0.0001) and night (nsyb/+, P<0.0049; Nf1RNAi/+, P<0.0036). N = 50–55. The median (solid line) as well as 25th and 75th percentiles (dotted lines) are shown. *p<0.05; ****p<0.0001. (TIF) [file pgen.1011049.s001.tif]

Supplemental Figure 2

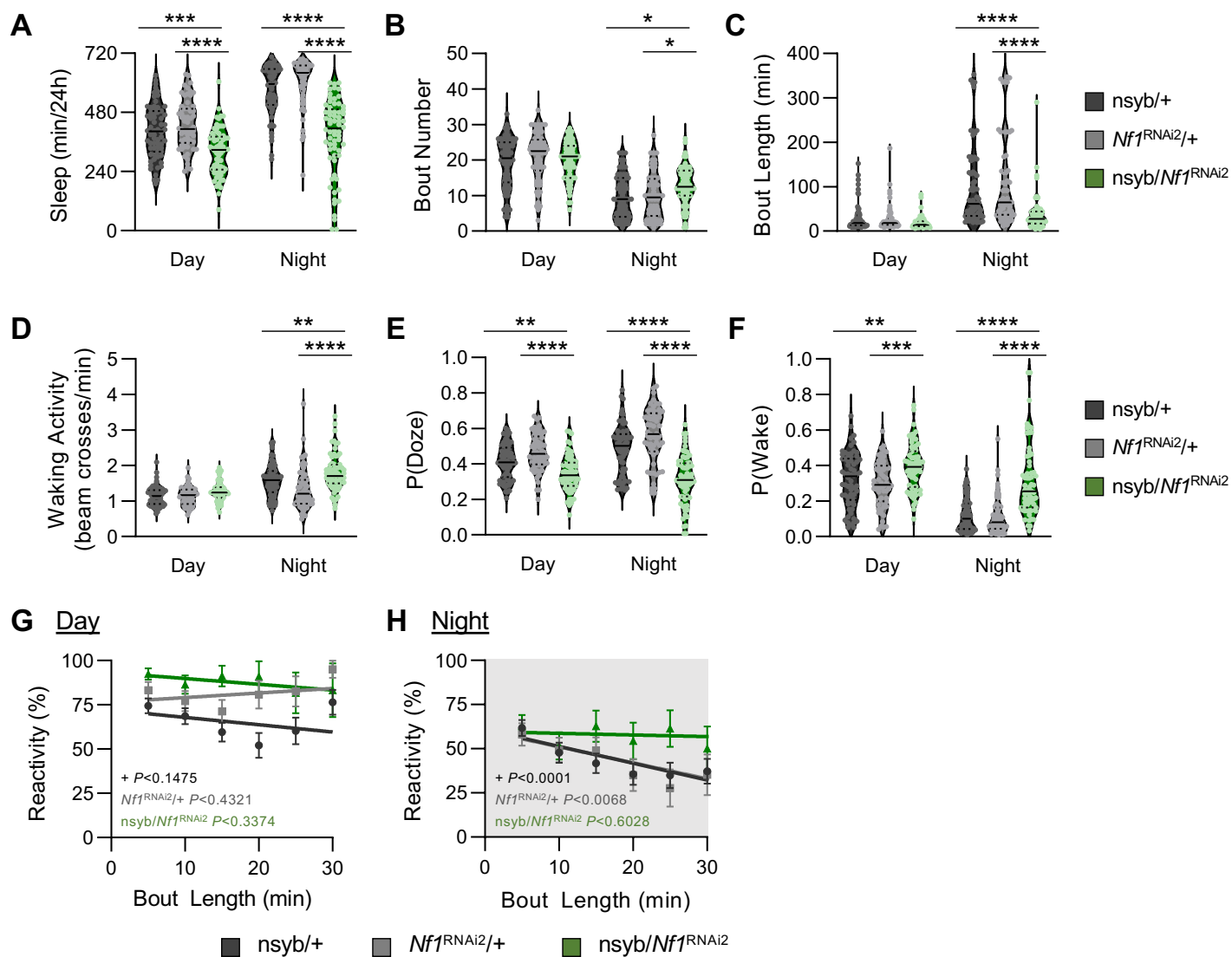

Supplement: S2 Fig — (A-F). Sleep and activity traits of pan-neuronal Nf1RNAi2 knockdown flies and their respective controls. A. There is a significant effect of genotype on sleep duration (two-way ANOVA: F2,378 = 79.47, P<0.0001). Compared to controls, pan-neuronal knockdown of Nf1 significantly reduces sleep during the day (nsyb/+, P<0.0001; Nf1RNAi2/+, P<0.0001) and night (nsyb/+, P<0.0001; Nf1RNAi2/+, P<0.0001). B. There is a significant effect of genotype on bout number (two-way ANOVA: F2,378 = 2.679, P<0.0499). Compared to controls, pan-neuronal knockdown of Nf1 significantly increases bout number during the night (nsyb/+, P<0.0195; Nf1RNAi2/+, P<0.0373), but not during the day (nsyb/+, P<0.8369; Nf1RNAi2/+, P<0.6346). C. There is a significant effect of genotype on bout length (two-way ANOVA: F2,378 = 18.02, P<0.0001). Compared to controls, pan-neuronal knockdown of Nf1 significantly reduces bout length during the ngiht (nsyb/+, P<0.0001; Nf1RNAi2/+, P<0.0001), but not during the day (nsyb/+, P<0.5054; Nf1RNAi2/+, P<0.6506). D. There is a significant effect of genotype on waking activity (two-way ANOVA: F2,378 = 17.32, P<0.0001). Compared to controls, pan-neuronal knockdown of Nf1 significantly increases waking activity during the day (nsyb/+, P<0.0035; Nf1RNAi/+, P<0.0001), but not during the day (nsyb/+, P<0.1695; Nf1RNAi/+, P<0.1646). E. There is a significant effect of genotype on the probability of falling asleep (two-way ANOVA: F2,378 = 71.99, P<0.0001). P(Doze) is significantly lower upon knockdown of Nf1 during the day (nsyb/+, P<0.0071; Nf1RNAi/+, P<0.0001) and night (nsyb/+, P<0.0001; Nf1RNAi/+, P<0.0001). F. There is a significant effect of genotype on the probability of waking up (two-way ANOVA: F2,378 = 41.99, P<0.0001). P(Wake) is significantly higher upon knockdown of Nf1 during the day (nsyb/+, P<0.0072; Nf1RNAi/+, P<0.0004) and night (nsyb/+, P<0.0001; Nf1RNAi/+, P<0.0001). N = 60–66. (G,H) Linear regression of (G) daytime and (H) nighttime reactivity as a functi [file pgen.1011049.s002.tif]

Supplemental Figure 3

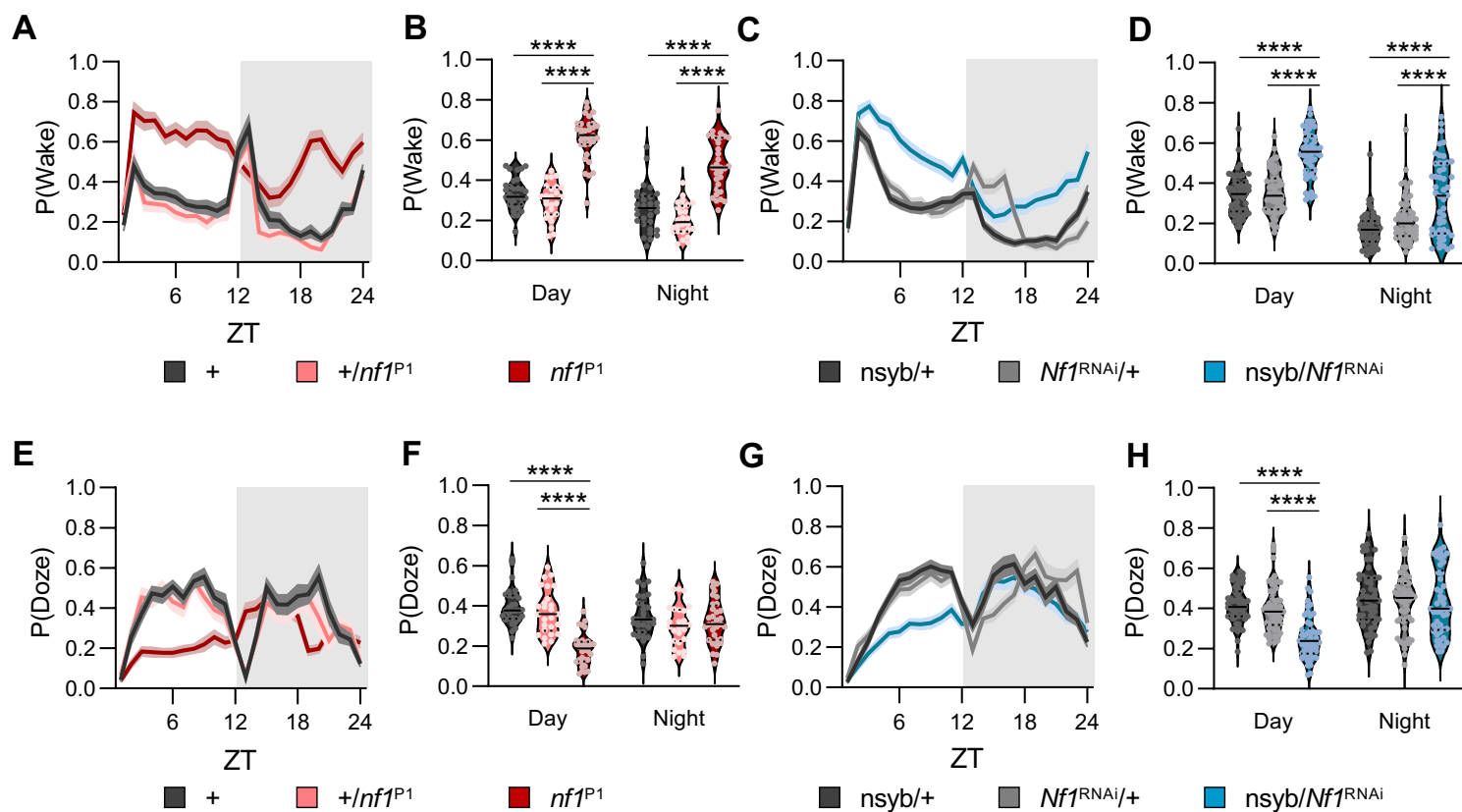

Supplement: S3 Fig — (A-D) Computational modeling of waking probabilities. A. Profiles of the probability of waking up in nf1P1 mutants, heterozygotes, and their control. B. There is a significant effect of genotype on the probability of waking up (two-way ANOVA: F2,172 = 144.6, P<0.0001). P(Wake) is significantly higher in nf1P1 mutant flies during the day (+, P<0.0001; het, P<0.0001) and night (+, P<0.0001; het, P<0.0001). C. Profiles of the probability of waking up in pan-neuronal Nf1RNAi knockdown flies and their controls. D. There is a significant effect of genotype on the probability of waking up (two-way ANOVA: F2,314 = 67.34, P<0.0001). P(Wake) is significantly higher upon knockdown of Nf1 during the day (nsyb/+, P<0.0001; nf1RNAi/+, P<0.0001) and night (nsyb/+, P<0.0001; Nf1RNAi/+, P<0.0001). N = 26–32. (E-H) Computational modeling of sleep probabilities. E. Profiles of the probability of falling asleep in nf1P1 mutants, heterozygotes, and their control. F. There is a significant effect of genotype on the probability of falling asleep (two-way ANOVA: F2,172 = 23.10, P<0.0001). P(Doze) is significantly lower in nf1P1 mutant flies during the day (+, P<0.0001; het, P<0.0001), but there is no difference during the night (+, P<0.0001; het, P<0.0001). G. Profiles of the probability of falling asleep in pan-neuronal Nf1RNAi knockdown flies and their controls. H. There is a significant effect of genotype on the probability of falling asleep (two-way ANOVA: F2,314 = 13.55, P<0.0001). P(Doze) is significantly lower upon knockdown of Nf1 during the day (nsyb/+, P<0.0001; Nf1RNAi/+, P<0.0001), but there is no difference during the night (nsyb/+, P<0.8053; Nf1RNAi/+, P<0.9999). N = 50–55. For profiles, shaded regions indicate ± SEM. White background indicates daytime, while gray background indicates nighttime. ZT indicates zeitgeber time. For violin plots, the median (solid line) as well as 25th and 75th percentiles (dotted lines) are shown. ****p<0.0001. (TIF) [file pgen.1011049.s003.tif]

Supplemental Figure 4

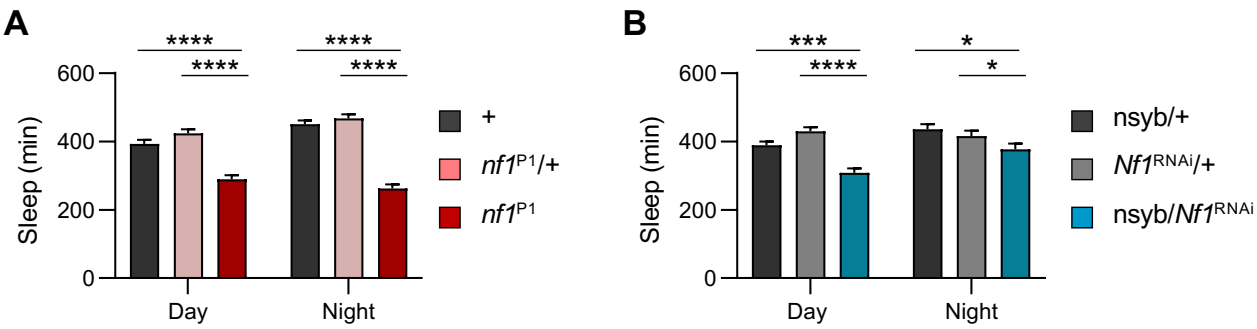

Supplement: S4 Fig — A. There is a significant effect of genotype on sleep duration (two-way ANOVA: F2,372 = 131.7, P<0.0001). Compared to control and heterozygote flies, nf1P1 mutants sleep significantly less during the day (+, P<0.0001; het, P<0.0001) and night (+, P<0.0001; het, P<0.0001). N = 58–70. B. There is a significant effect of genotype on sleep duration (two-way ANOVA: F2,220 = 19.05, P<0.0001). Compared to controls, pan-neuronal knockdown of Nf1 significantly reduces sleep during the day (nsyb/+, P<0.0003; Nf1RNAi/+, P<0.0001) and night (nsyb/+, P<0.0106; Nf1RNAi/+, P<0.0135). N = 36–41. The median (solid line) as well as 25th and 75th percentiles (dotted lines) are shown. *p<0.05; ***p<0.001; ****p<0.0001. (TIF) [file pgen.1011049.s004.tif]

Supplemental Figure 5

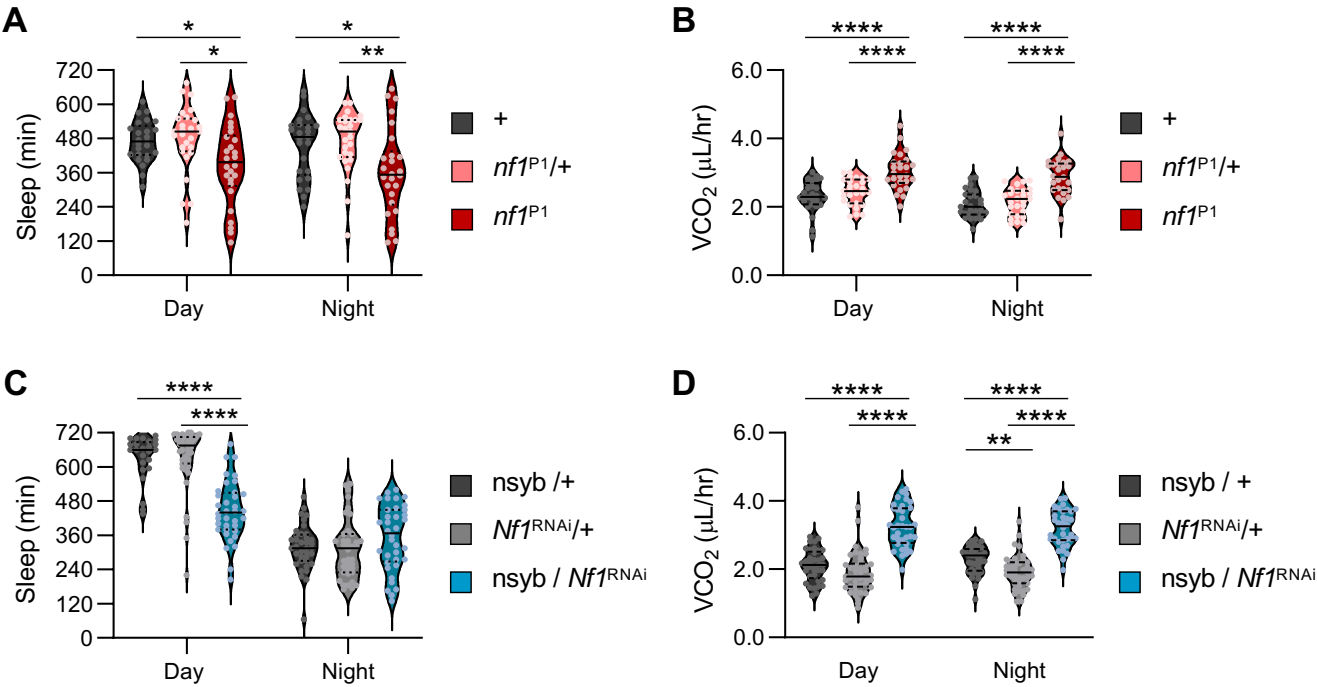

Supplement: S5 Fig — Sleep duration and metabolic rate were measured in the SAMM system. A. There is a significant effect of genotype on sleep duration (two-way ANOVA: F2,154 = 10.92, P<0.0001). Compared to control and heterozygote flies, nf1P1 mutants sleep significantly less during the day (+, P<0.0414; het, P<0.0159) and night (+, P<0.0167; het, P<0.0033). B. There is a significant effect of genotype on metabolic rate (two-way ANOVA: F2,154 = 43.72, P<0.0001). Compared to control and heterozygote flies, nf1P1 mutants significantly increase CO2 output during the day (+, P<0.0001; het, P<0.0001) and night (+, P<0.0001; het, P<0.0001). N = 26–27. C. There is a significant effect of genotype on sleep duration (two-way ANOVA: F2,224 = 13.79, P<0.0001). Compared to controls, pan-neuronal knockdown of Nf1 significantly decreases sleep during the day (nsyb/+, P<0.0001; Nf1RNAi/+, P<0.0001), but not the night (nsyb/+, P<0.3333; Nf1RNAi/+, P<0.2203). D. There is a significant effect of genotype on metabolic rate (two-way ANOVA: F2,224 = 136.0, P<0.0001). Compared to controls, pan-neuronal knockdown of Nf1 significantly increases CO2 output during the day (nsyb/+, P<0.0001; Nf1RNAi/+, P<0.0001) and night (nsyb/+, P<0.0001; Nf1RNAi/+, P<0.0001). N = 30–44. The median (solid line) as well as 25th and 75th percentiles (dotted lines) are shown. *p<0.05; **p<0.01; ****p<0.0001. (TIF) [file pgen.1011049.s005.tif]

Supplemental Figure 6

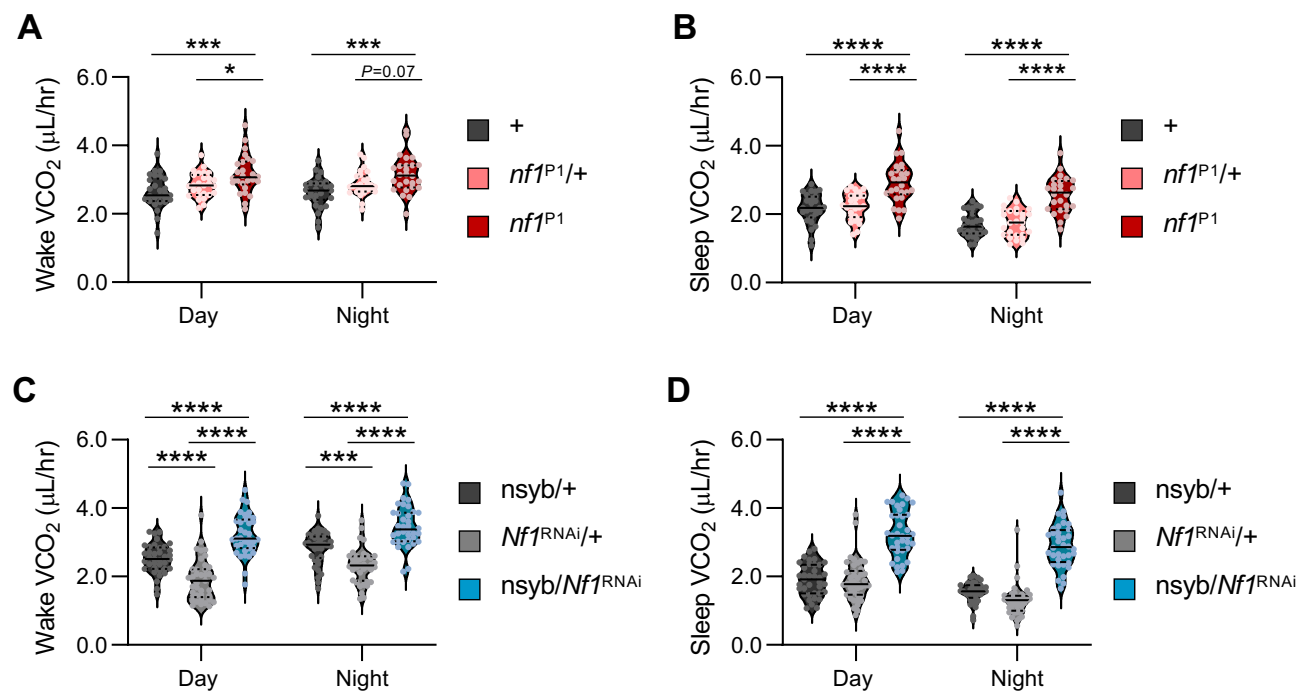

Supplement: S6 Fig — A. There is a significant effect of genotype on metabolic rate during waking (two-way ANOVA: F2,154 = 16.76, P<0.0001). In the daytime, nf1P1 mutants significantly increase waking CO2 output compared to control and heterozygote flies (+, P<0.0003; het, P<0.0412). At night, nf1P1 mutants significantly increase waking CO2 output compared to control flies (+, P<0.0002), with heterozygotes being intermediate (het, P<0.0762). B. There is a significant effect of genotype on metabolic rate during sleep (two-way ANOVA: F2,154 = 53.48, P<0.0001). Compared to control and heterozygote flies, nf1P1 mutants significantly increase CO2 output during sleep during the day (+, P<0.0001; het, P<0.0001) and night (+, P<0.0001; het, P<0.0001). N = 26–27. C. There is a significant effect of genotype on metabolic rate during waking (two-way ANOVA: F2,224 = 97.10, P<0.0001). Compared to controls, pan-neuronal knockdown of Nf1 significantly increases waking CO2 output during the day (nsyb/+, P<0.0001; Nf1RNAi/+, P<0.0001) and night (nsyb/+, P<0.0001; Nf1RNAi/+, P<0.0001). D. There is a significant effect of genotype on metabolic rate during sleep (two-way ANOVA: F2,224 = 176.1, P<0.0001). Compared to controls, pan-neuronal knockdown of Nf1 significantly increases CO2 output during sleep during the day (nsyb/+, P<0.0001; Nf1RNAi/+, P<0.0001) and night (nsyb/+, P<0.0001; Nf1RNAi/+, P<0.0001). N = 30–44. The median (solid line) as well as 25th and 75th percentiles (dotted lines) are shown. *p<0.05; ***p<0.001; ****p<0.0001. (TIF) [file pgen.1011049.s006.tif]

Supplemental Figure 7

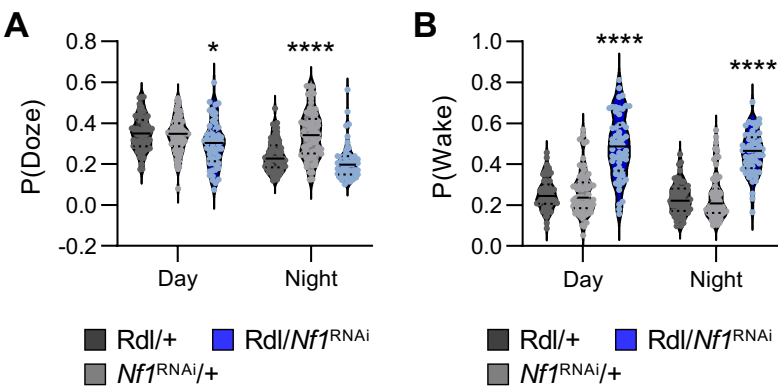

Supplement: S7 Fig — A. There is a significant effect of genotype on the probability of falling asleep (two-way ANOVA: F2,362 = 27.27, P<0.0001). Compared to controls, knockdown of Nf1 in Rdl-expressing neurons significantly reduces P(Dose) during the day (Rdl/+, P<0.0097; Nf1RNAi/+, P<0.0372), but only compared to one control during the night (Rdl/+, P<0.1412; Nf1RNAi/+, P<0.0001). B. There is a significant effect of genotype on the probability of waking up (two-way ANOVA: F2,362 = 161.5, P<0.0001). Compared to controls, knockdown of Nf1 in Rdl-expressing neurons significantly increases P(Wake) and occurs during the day (Rdl/+, P<0.0001; Nf1RNAi/+, P<0.0001) and night (Rdl/+, P<0.0001; Nf1RNAi/+, P<0.0001). N = 57–66. The median (solid line) as well as 25th and 75th percentiles (dotted lines) are shown. *p<0.05; ****p<0.0001. (TIF) [file pgen.1011049.s007.tif]

Supplemental Figure 8

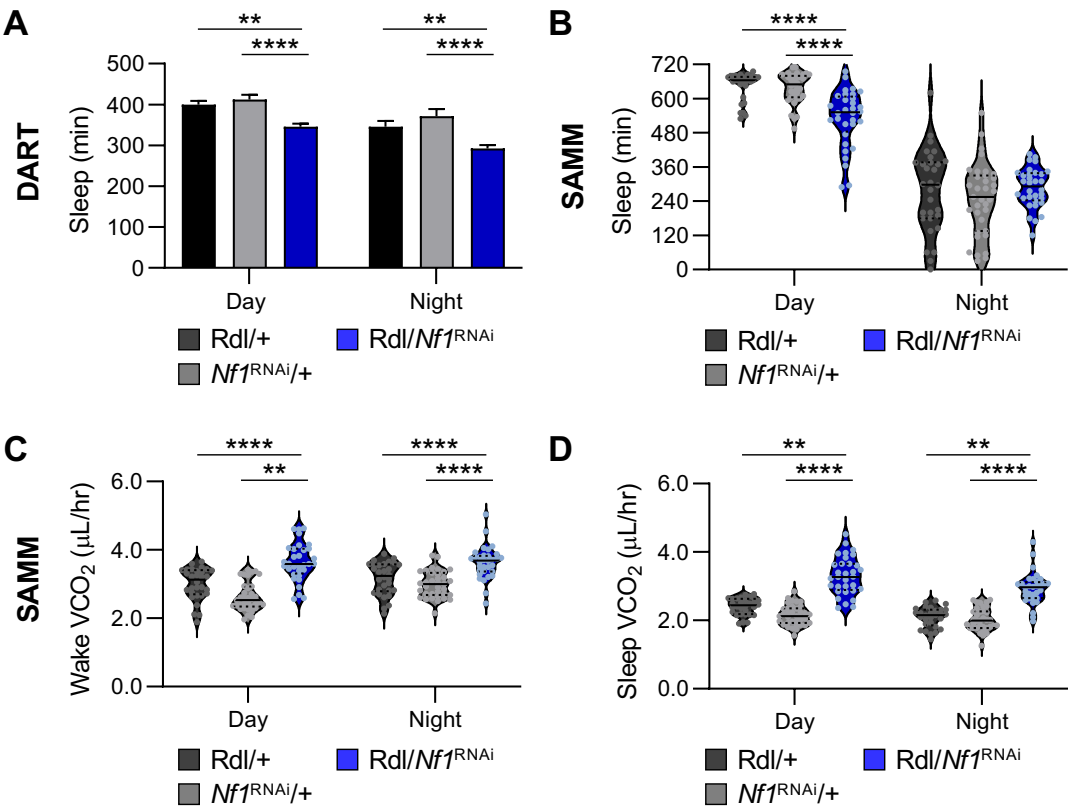

Supplement: S8 Fig — A. There is a significant effect of genotype on sleep duration in the DART system (two-way ANOVA: F2,378 = 27.38, P<0.0001). Compared to controls, knockdown of Nf1 in Rdl-expressing neurons significantly reduces sleep and occurs during day (Rdl/+, P<0.0012; Nf1RNAi/+, P<0.0001) and night (Rdl/+, P<0.0015; Nf1RNAi/+, P<0.0001). N = 49–61. B. There is a significant effect of genotype on sleep duration in the SAMM system (two-way ANOVA: F2,188 = 4.708, P<0.0101). Compared to controls, knockdown of Nf1 in Rdl-expressing neurons significantly reduces sleep, but only occurs during the day (Rdl/+, P<0.0001; Nf1RNAi/+, P<0.0001) and not the night (Rdl/+, P<0.9410; Nf1RNAi/+, P<0.2371). C. There is a significant effect of genotype on metabolic rate during waking (two-way ANOVA: F2,188 = 54.14, P<0.0001). Compared to controls, knockdown of Nf1 in Rdl-expressing neurons significantly increases CO2 output during the day (Rdl/+, P<0.0001; Nf1RNAi/+, P<0.0001) and night (Rdl/+, P<0.0003; Nf1RNAi/+, P<0.0001). D. There is a significant effect of genotype on metabolic rate during sleep (two-way ANOVA: F2,188 = 136.1, P<0.0001). Compared to controls, knockdown of Nf1 in Rdl-expressing neurons significantly increases CO2 output during the day (Rdl/+, P<0.0001; Nf1RNAi/+, P<0.0001) and night (Rdl/+, P<0.0001; Nf1RNAi/+, P<0.0001). N = 30–35. The median (solid line) as well as 25th and 75th percentiles (dotted lines) are shown. **p<0.01; ****p<0.0001. (TIF) [file pgen.1011049.s008.tif]

Supplemental Figure 9

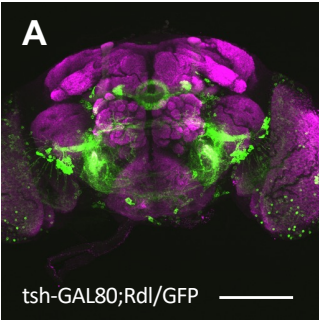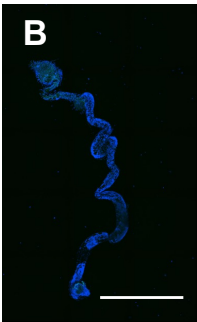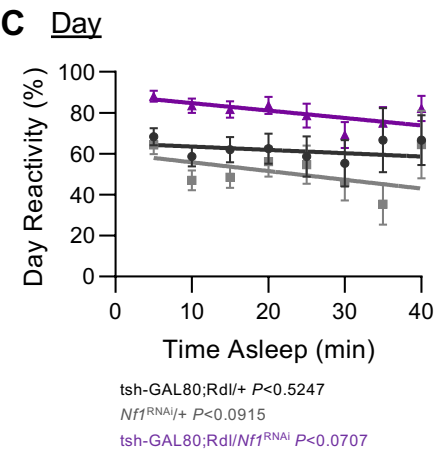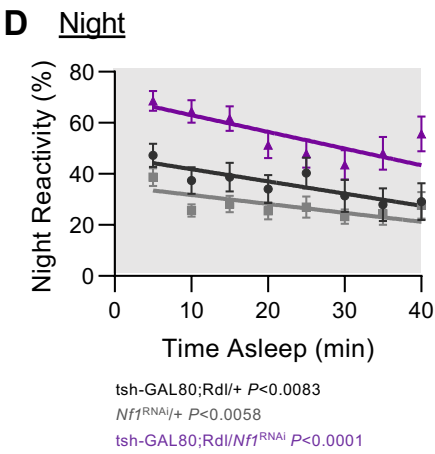

Supplement: S9 Fig — The tsh-GAL80 repressor was used to restrict expression of Nf1RNAi to GABAA receptor neurons in the brain. (A,B). The expression pattern of tsh-GAL80; Rdl-GAL4 neurons is visualized with GFP in the brain (A) and the gut (B). For the brain, background staining is NC82 antibody (magenta). Scale bar = 100μm. For the gut, background staining is DAPI (blue). Scale bar = 1000μm. (C,D) Measurements of reactivity in Nf1RNAi knockdown flies and their respective controls using the DART system. C. Linear regression of daytime reactivity as a function of time asleep in Nf1RNAi knockdown flies and their controls. The intercepts of each regression line are significantly different from each other (F2,1416 = 71.71, P<0.0001). D. Linear regression of nighttime reactivity as a function of time asleep in Nf1RNAi knockdown flies and their controls. The intercepts of each regression line are significantly different from each other (F2,1724 = 76.56, P<0.0001). N = 52–71. Error bars indicate ± SEM. The P-values in each panel indicate whether the slope of the regression line is significantly different from zero. White background indicates daytime, while gray background indicates nighttime. (TIF) [file pgen.1011049.s009.tif]

Supplemental Figure 10

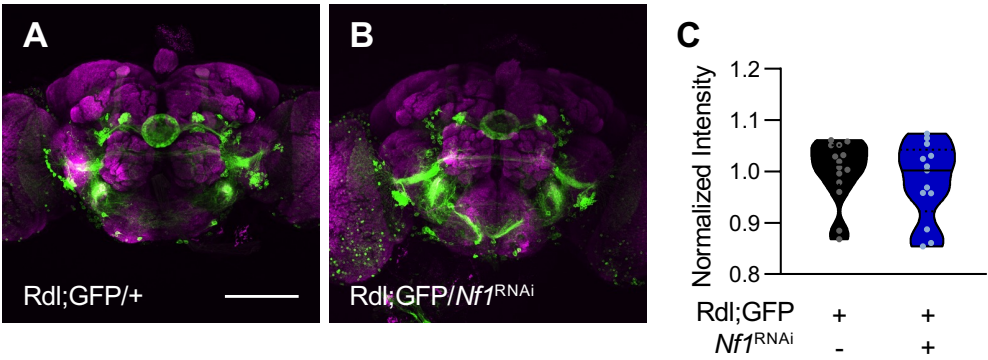

Supplement: S10 Fig — GABAA receptor neurons were targeted using the Rdl-GAL4 driver. (A,B). The expression pattern of Rdl-expressing neurons is visualized with GFP. Background staining is NC82 antibody (magenta). Scale bar = 100μm. C. Knockdown of Nf1 in Rdl-expressing neurons has no effect on fluorescence intensity (t-test: t25 = 0.7508, P<0.4598). The median (solid line) as well as 25th and 75th percentiles (dotted lines) are shown. (TIF) [file pgen.1011049.s010.tif]

Supplemental Figure 11

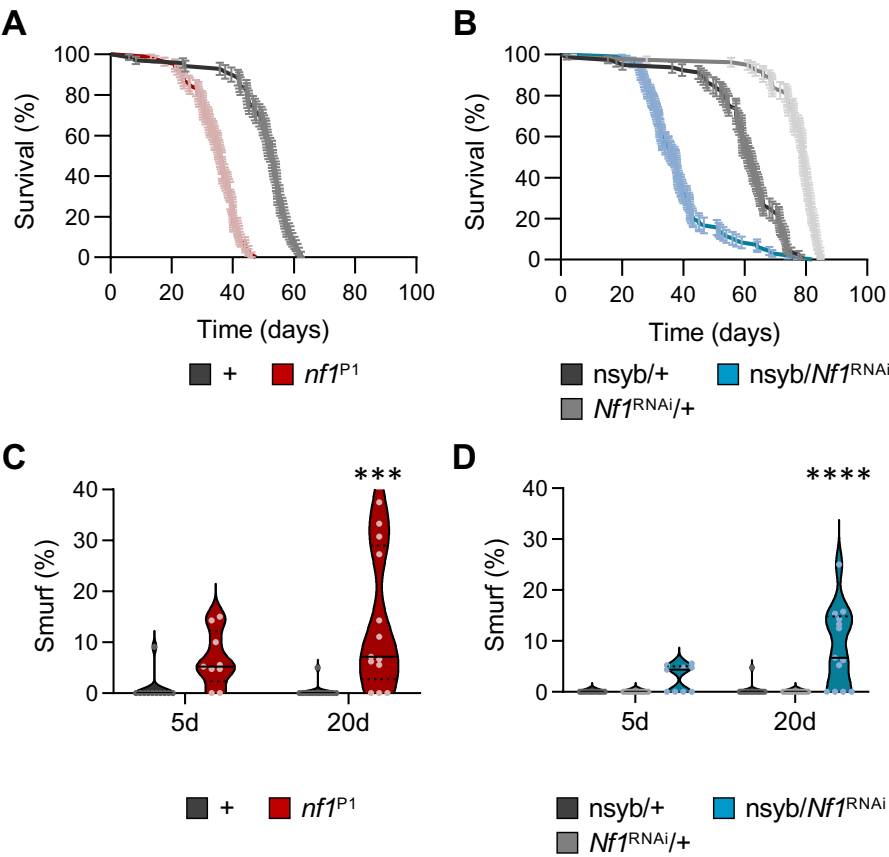

Supplement: S11 Fig — A. Compared to control flies, loss of Nf1 significantly decreases longevity (Log-Rank test: χ2 = 209.0, d.f. = 2, P<0.0001). N = 70–84. B. Compared to controls, pan-neuronal knockdown of Nf1 significantly decreases longevity (Log-Rank test: χ2 = 253.4, d.f. = 2, P<0.0001). N = 80–96. C. There is a significant effect of genotype on intestinal permeability (two-way ANOVA: F1,42 = 29.45, P<0.0002). Loss of Nf1 does not change intestinal barrier dysfunction in 5d flies (+, P<0.0565; het, P<0.0648), but significantly increases in 20d flies (+, P<0.0001; het, P<0.0001). N = 9–13. D. There is a significant effect of genotype on intestinal permeability (two-way ANOVA: F2,65 = 18.80, P<0.0001). Pan-neuronal knockdown of Nf1 does not change intestinal barrier dysfunction in 5d flies (nsyb/+, P<0.2093; Nf1RNAi/+, P<0.1973), but significantly increases in 20d flies (nsyb/+, P<0.0001; Nf1RNAi/+, P<0.0001). N = 9–12. The median (solid line) as well as 25th and 75th percentiles (dotted lines) are shown. ***p<0.001; ****p<0.0001. (TIF) [file pgen.1011049.s011.tif]

Supplemental Figure 12

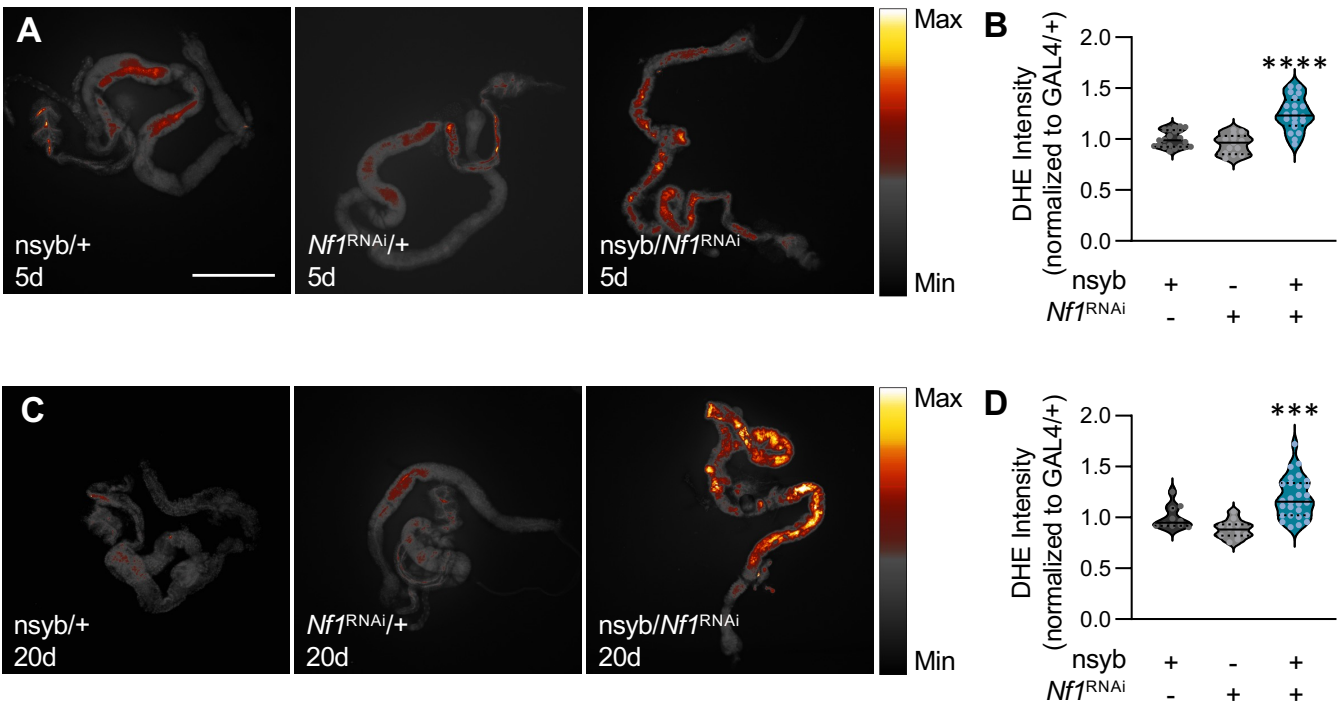

Supplement: S12 Fig — ROS was measured in 5d and 20d flies by quantifying oxidized DHE levels. A. Oxidized DHE was measured in 5d control and Nf1 knockdown flies. B. Pan-neuronal knockdown of Nf1 significantly increases oxidized DHE signal intensity in 5d flies (one-way ANOVA: F2,51 = 30.37, P<0.0001). N = 13–21. C. Oxidized DHE was measured in 20d control and Nf1 knockdown flies. D. Pan-neuronal knockdown of Nf1 significantly increases oxidized DHE signal intensity in 20d flies (one-way ANOVA: F2,47 = 16.36, P<0.0001). N = 10–28. Scale bar = 500μm. The median (solid line) as well as 25th and 75th percentiles (dotted lines) are shown. ***p<0.001; ****p<0.0001. (TIF) [file pgen.1011049.s012.tif]

Supplemental Figure 13

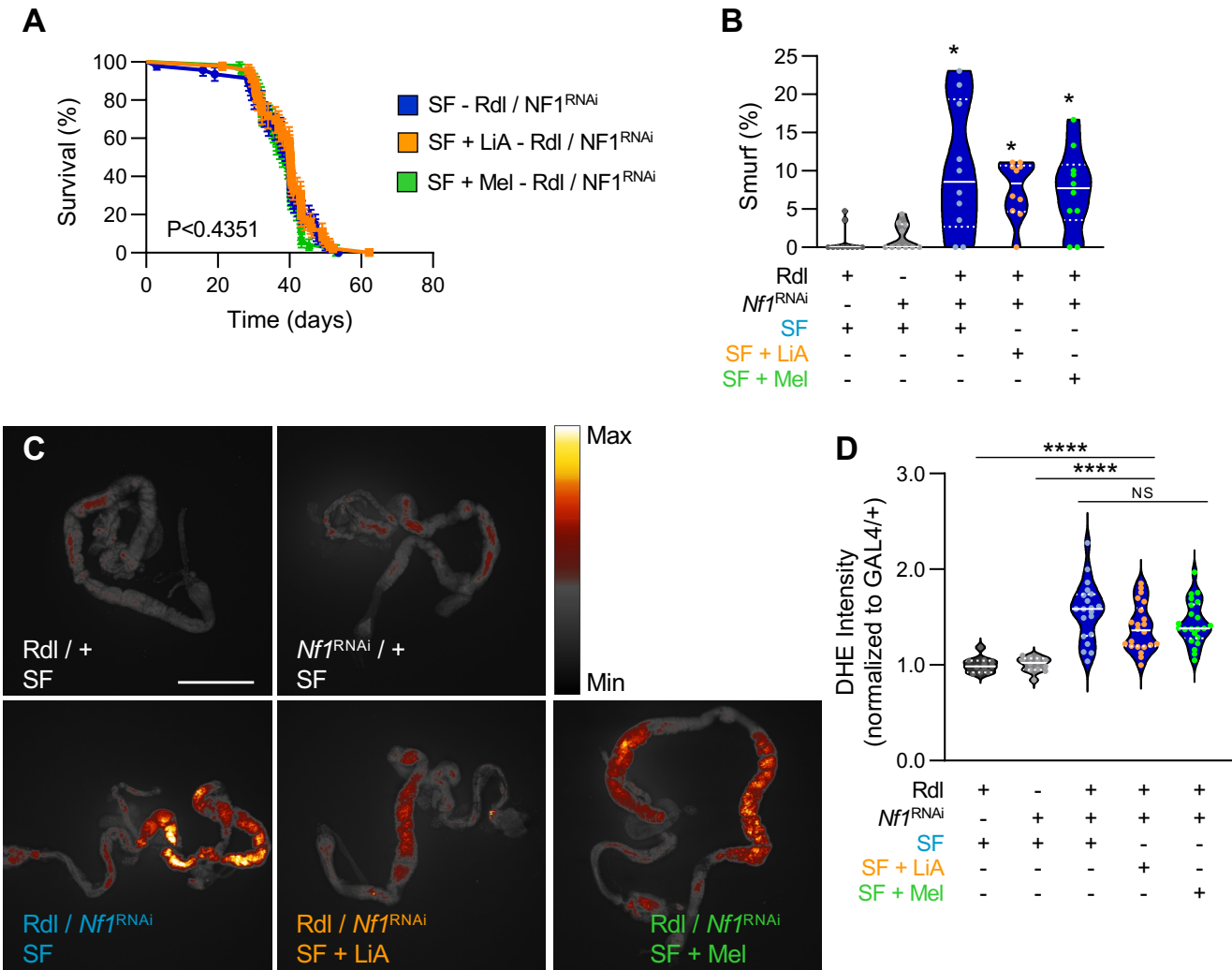

Supplement: S13 Fig — The antioxidants Lipoic Acid (LiA) or Melatonin (Mel) were added to standard food. A. Antioxidant feeding has no effect on longevity in Rdl-GAL4/Nf1RNAi flies (Log-Rank test: χ2 = 1.665, d.f. = 2, P<0.4351). N = 46–48. B. There is significant effect of genotype on intestinal permeability in 20d flies upon knockdown of Nf1 in Rdl-expressing neurons (one-way ANOVA: F4,37 = 7.634, P<0.0001), but no effect of antioxidant feeding among Nf1-deficient flies (one-way ANOVA: F2,19 = 0.7633, P<0.4799). N = 11–22. C. Oxidized DHE was measured in 20d flies fed either standard food or standard food with antioxidants. Scale bar = 500μm. D. There is significant effect of genotype on DHE signal intensity in 20d flies upon knockdown of Nf1 in Rdl-expressing neurons (one-way ANOVA: F4,82 = 18.02, P<0.0001), but no effect of antioxidant feeding among Nf1-deficient flies (one-way ANOVA: F2,61 = 2.2682, P<0.1122). N = 11–22. For survival measurements, error bars indicate ± SEM. For gut measurements, the median (solid line) as well as 25th and 75th percentiles (dotted lines) are shown. *p<0.05; ****p<0.0001. (TIF) [file pgen.1011049.s013.tif]

Supplemental Figure 14

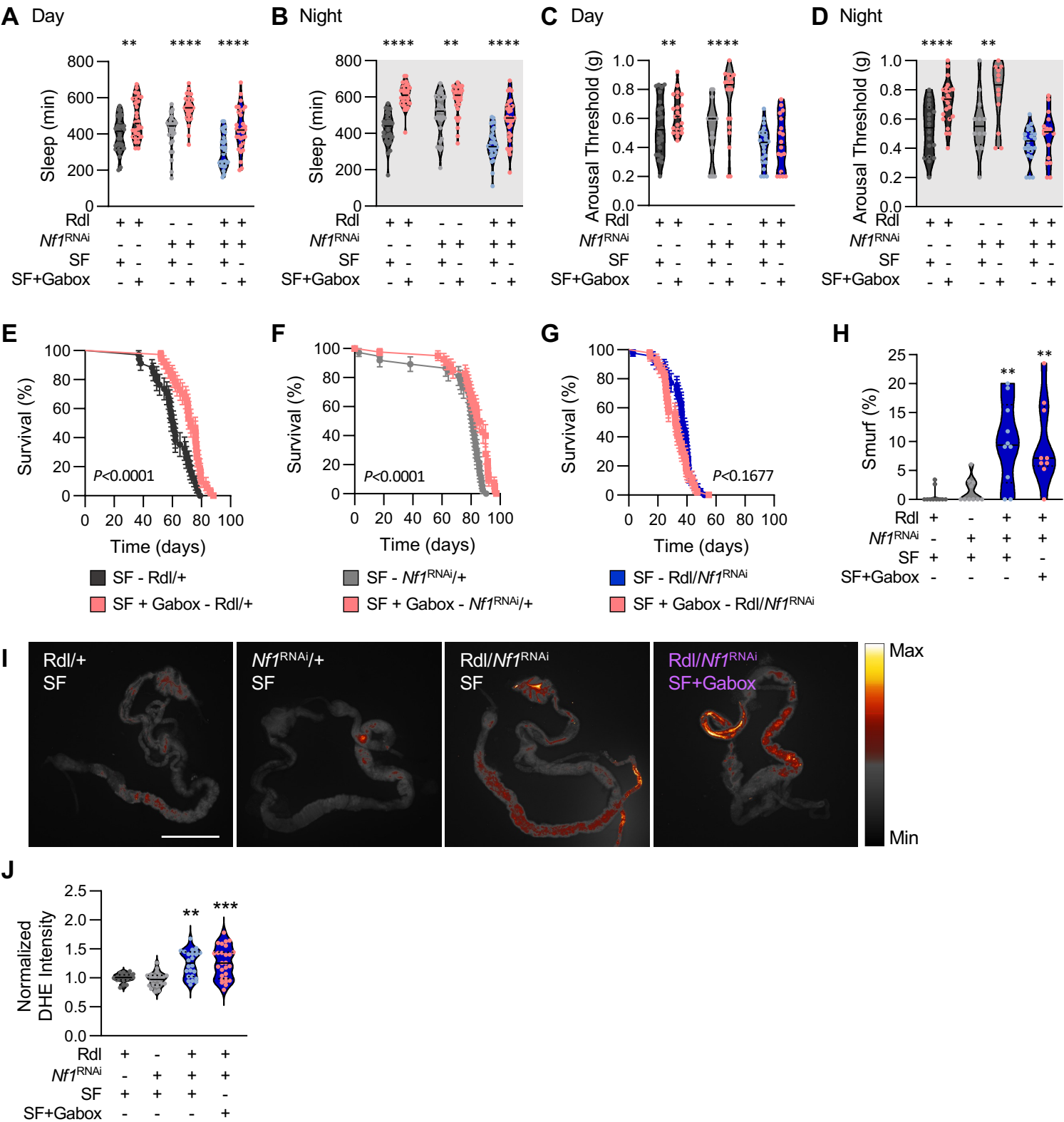

Supplement: S14 Fig — Gaboxadol (Gabox; 0.1 mg/mL) was added to the fly diet to promote sleep. A. There is a significant effect of gaboxadol on daytime sleep duration (two-way ANOVA: F1,182 = 59.14, P<0.0001). Gaboxadol increases daytime sleep in all genotypes tested (Rdl/+, P<0.0023; Nf1RNAi/+, P<0.0001; Rdl/Nf1RNAi, P<0.0001). B. There is a significant effect of gaboxadol on nighttime sleep duration (two-way ANOVA: F1,182 = 92.63, P<0.0001). Gaboxadol increases nighttime sleep in all genotypes tested (Rdl/+, P<0.0001; Nf1RNAi/+, P<0.0018; Rdl/Nf1RNAi, P<0.0001). N = 30–32. C. There is a significant effect of gaboxadol on daytime arousal threshold (REML: F1,155 = 15.41, P<0.0001). Gaboxadol increases daytime arousal threshold in control flies, but not in flies with knockdown of Nf1 in Rdl-expressing neurons (Rdl/+, P<0.0080; Nf1RNAi/+, P<0.0001; Rdl/Nf1RNAi, P<0.5755). D. There is a significant effect of gaboxadol on nighttime arousal threshold (REML: F1,132 = 30.44, P<0.0001). Gaboxadol increases nighttime arousal threshold in control flies, but not in flies with knockdown of Nf1 in Rdl-expressing neurons (Rdl/+, P<0.0001; Nf1RNAi/+, P<0.0029; Rdl/Nf1RNAi, P<0.4634). N = 20–34. E. Gaboxadol significantly extends longevity in control Rdl-GAL4/+ flies (Log-Rank test: χ2 = 15.36, d.f. = 1, P<0.0001). N = 34–37. F. Gaboxadol significantly extends longevity in control Nf1RNAi/+ flies (Log-Rank test: χ2 = 15.51, d.f. = 1, P<0.0001). N = 37–40. G. Gaboxadol has no effect on longevity in Rdl-GAL4/ Nf1RNAi flies (Log-Rank test: χ2 = 1.904, d.f. = 1, P<0.1677). N = 43–44. H. Gaboxadol has no effect on intestinal permeability in 20d flies with knockdown of Nf1 in Rdl-expressing neurons (one-way ANOVA: F3,35 = 9.357, P<0.0001). N = 9–10. (I,J) ROS was measured in 20d flies by quantifying oxidized DHE. I. Oxidized DHE was measured in 20d flies fed either standard food or standard food with Gaboxadol. Scale bar = 500μm. J. Gaboxadol has no effect on oxidized DHE signal intensity in 20d flies with kn [file pgen.1011049.s014.tif]
