# Supplementary material for: Neurofibromin 1 mediates sleep depth in Drosophila
Source: PLoS Genet. 2023 Dec 13;19(12):e1011049. doi: 10.1371/journal.pgen.1011049 (PMC10763969; doi:10.1371/journal.pgen.1011049)
Supplement: Attachment — Submitted filename: Reviewer Comments V4.docx [file pgen.1011049.s016.docx]

**Reviewer Comments**

We thank the reviewers for their thorough review and thoughtful feedback on our manuscript. This revised version includes significant revisions of the text, analyses, and additional experiments. We believe the manuscript has been significantly strengthened and the implications are clearer. We provide a detailed response to individual suggestions below.

**Reviewer #1**

**This manuscript from Brown et al. examines the consequences of Nf1 disruption on sleep time, architecture, and function. Interestingly, Nf1 mutants and RNAi-mediated knock-down flies show reduced sleep time, fragmented sleep architecture, and weakened reductions in metabolic rate during prolonged sleep episodes. The observations of locomotion, arousal responses, and metabolic rate in these studies provides a comprehensive picture of sleep/wake phenotypes in these flies and is a notable strength of the paper. The same genotypes of Nf1-deficient flies also show decreased lifespan, increased gut permeability, and increased oxidative stress in the gut. Together, these data points are consistent with the hypothesis that flies lacking Nf1 are deficient in consolidated, deep sleep that may contribute to gut homeostasis and clearance of oxidative stress.**

Thank you for these supportive comments.

**While these data indicate a strong correlation between sleep and gut phenotypes, Nf1 seems to be pleiotropic and additional data that directly links the gut and sleep issues would greatly strengthen the manuscript. Testing gut permeability and oxidative stress in Nf1-deficient flies after increasing sleep either pharmacologically or genetically might allow for a direct test of whether sleep loss contributes to these phenotypes.**

We appreciate these suggestions, and fully agree that these experiments are needed to strengthen our conclusions. We have now performed additional experiments where we restore sleep to Rdl>*Nf1*^RNAi^ flies by treating them with the GABA_A_ Receptor agonist Gaboxadol (Fig S13). We found that Gaboxadol increases sleep in both control and *Nf1* deficient flies, but a concomitant increase in arousal threshold only occurred in the controls. We also found that while Gaboxadol increases longevity in control flies, it does not impact longevity, gut permeability, or gut ROS. These results support the notion that the deficiency in lifespan and gut homeostasis are not due to loss of sleep duration, but may derive from the loss of sleep quality as measured by arousal threshold. We now describe these findings in the Results (Line 248).

**Additionally, it would be useful to image the expression pattern of Rdl-Gal4 in the gut - it is possible that Nf1 RNAi using this driver may be having local effects in the periphery in addition to the brain.**

We now include an image of *Rdl* expression in the gut, where we find a small amount of GFP signal innervating the ampula of the intestine (Fig 3A). To confirm this finding, we have imaged *Rdl*-GAL4>UAS-mCD8:GFP flies containing tsh-GAL80, which inhibits GAL4-induced transgene expression in the thoracic and abdominal nervous system (Fig S9A-B). We find that the presence of tsh-GAL80 abolishes *Rdl* expression in the gut. Further, day and night reactivity remain elevated in *Nf1*-knockdown flies harboring *tsh*-GAL80, confirming that the effects derive from *Nf1* expression within the brain (Fig S9C-D). We now describe these findings in the Results (Line 190).

**Other points:**

**1. Sleep bout data in Figures 1, 4, and S2, along with waking activity (Figs. S1, S2 ) and P(wake) and P(doze) data (Figs. S2, S7) should display day and night values separately, just as sleep time data is organized throughout the manuscript.**

Thank you for this suggestion. We have now modified figures throughout the manuscript to display both day and night values, amended the figure legends to reflect the appropriate statistical analysis, and changed the text in the results section when appropriate.

**2. The quantification of Reactivity % in Figure 2E-H is unclear - did these experiments use a fixed stimulus intensity? Or does the stimulus intensity vary as in the arousal threshold trials?**

Indeed, these experiments used a fixed stimulus intensity. This is consistent with previous manuscripts using these metrics from our labs and others (Ferguson et al 2017; Troup et al 2018; Brown et al 2020). We have added additional detail in the Methods, and references within the manuscript. We now state: “Reactivity was assessed at the maximum stimulus intensity of 1.2 g, with an inter-stimulus delay of 15 s, once per hour over 24 hours starting at ZT0. Measurements of reactivity are reported as the percentage of sleeping flies that are responsive to the stimulus” (Line 449).

**3. Were gut dissections performed at a particular time of day for the DHE staining to measure oxidative stress?**

Yes. Gut dissections were performed during ZT 0-2. We have modified the text to include this detail, and included additional clarifications within the methods section (Line 509).

**4. While lifespan is shortened in some short-sleeping flies as the authors describe (lines 202-3 and 302-3), a few other genotypes with severe sleep reductions do not seem to have reduced lifespan - eg. fumin mutants (Kume, 2005; J Neurosci) and pan-neuronal insomniac RNAi flies (Stavropoulos & Young, 2011; Neuron). It might be worth mentioning these findings in the discussion of lifespan in short-sleeping flies.**

Thank you for these suggestions. We have included this discussion and references within the manuscript. We think this is particularly relevant given our new findings suggest that arousal threshold, rather than total sleep duration, are associated with the changes in lifespan. We now state in the Discussion: “However, there are many examples of short sleeping genetic mutants, as well as in artificially selected short-sleeping flies and chronically sleep deprived wildtype flies where lifespan is not impacted (Geissmann et al., 2019; Harbison et al., 2017; Kume et al., 2005; Stavropoulos & Young, 2011)” (Line 352).

**5. The coincidence of elevated oxidative stress and increased gut permeability in Nf1 knock-down flies suggests that the two might be linked. Would feeding these flies antioxidants reduce/delay the occurrence of smurfed flies or extend lifespan?**

Thank you for this suggestion. We have tried our best to perform both experiments within the reasonable time frame provided. In our hands, feeding flies antioxidants trended towards a reduction in gut ROS, but the results were statistically insignificant (**See Figure 1 below**). We also did not observe a change in longevity or intestinal permeability. We believe this approach needs further optimization in our hands, and therefore we prefer to leave these inconclusive results out of the manuscript. Conversely, we found that dietary restriction extends longevity, reduces intestinal permeability, and decreases ROS levels in *Nf1*-defficient flies (Fig 5). The effects on intestinal permeability and ROS restore function to near-control levels. These findings support the notion that these processes are linked, and raise the possibility that the gut phenotypes may relate to metabolic changes previously identified in *Nf1* mutants. We now describe these findings in the Results (Line 261) and describe the implications of these findings in the discussion (Line 379).

**Reviewer #2**

**The role of NF1 in sleep in flies has been studied my several groups. In this paper, the authors revisit the role of NF1 in sleep, focusing on the regulation of sleep depth. Interesting findings are: (1) NF1 loss-of-function mutants, pan-neuronal Nf1 knockdown, and Nf1 knockdown with GABA_A receptor (Rdl)-GAL4, all three genotypes of flies show reduction in sleep depth. (2) All these flies also show increased metabolic rate. Although these are interesting observations, since deep sleep causes metabolic rate reduction as authors also acknowledge (more precisely, reduced metabolic rate is a signature of the deep sleep state), (1) and (2) are the description of the same phenomenon, and this relationship is not specific to NF1 loss of function. In this sense, the novelty of this paper is limited. This study does not address the molecular or neuronal mechanism underpinning the role of NF1 in sleep depth /metabolic regulation either. Therefore, the work is deemed preliminary, and the results presented do not support their main conclusion. Below are some comments and suggestions that may improve the manuscript.**

**Figure 1. Antioxidant feeding has no effect on lifespan or gut homeostasis in *Nf1*-deficient flies.** The antioxidants Lipoic Acid (LiA) or Melatonin (Mel) were added to standard food. **A.** Antioxidant feeding has no effect on longevity in Rdl-GAL4/*Nf1*^RNAi^ flies (Log-Rank test: χ^2^=1.665, d.f.=2, *P*<0.4351). N=46-48. **B.** There is significant effect of genotype on intestinal permeability in 20d flies upon knockdown of *Nf1* in *Rdl*-expressing neurons (one-way ANOVA: F_4,37_ = 7.634, *P*<0.0001), but no effect of antioxidant feeding among *Nf1*-deficient flies (one-way ANOVA: F_2,19_ = 0.7633, *P*<0.4799). N=11-22. **C.** Oxidized DHE was measured in 20d flies fed either standard food or standard food with antioxidants. Scale bar = 500μm. **D.** There is significant effect of genotype on DHE signal intensity in 20d flies upon knockdown of *Nf1* in *Rdl*-expressing neurons (one-way ANOVA: F_4,82_ = 18.02, *P*<0.0001), but no effect of antioxidant feeding among *Nf1*-deficient flies (one-way ANOVA: F_2,61_ = 2.2682, *P*<0.1122). N=11-22. For survival measurements, error bars indicate ± SEM. For gut measurements, the median (solid line) as well as 25th and 75th percentiles (dotted lines) are shown. **p*<0.05; *****p*<0.0001.

We appreciate these comments. We note that elevated metabolic rate can occur independently of sleep regulation. We view the finding that *Nf1* mutant flies do not enter deep sleep, based on multiple criteria, to be significant. We also note that this is the first manuscript to identify gut dysfunction in *Nf1* mutants, and to link it to sleep. We have now included multiple additional data sets, as well as clarifications within the text, that we hope address concerns of novelty.

**Major comments**

**1. As summarized above, the novelty of this study is obscure. In particular, data shown in Fig. 1 and Supplemental Figs 1-2 are essentially the reproduction of the published study (King et al, G3 2016). It is nice to see the reproducibility of the results, but it does not add new insights.**

We appreciate the reference to King et al, 2016. This manuscript identified decreased sleep and hyperactivity in *Nf1* mutant flies, and this was attributed to excessive grooming. However, this manuscript does not examine sleep fragmentation. In these figures, we perform a more detailed analysis of sleep, examining arousal threshold, sleep drive, and sleep-dependent changes in metabolic rate. As far as we know, there is no identified mutant with the *Nf1* phenotypes we have defined here.. We agree that Figure 1, on its own, appears to lack novelty. Therefore, we have combined it with Figure 2 in this revised version and hope this does a better job of highlighting the novel aspects of the manuscript..

**2. Arousal threshold assay with the DART system (Fig. 2C and D) shows reduced arousal threshold both during the day and night in NF1 mutants and NF1 pan-neuronal knockdown, but the reactivity after the sleep period does not change during the day (Fig. 2E -H). I do not understand why the results of these two assays are discrepant. Adding more detailed explanation on Fig. 2A and B would also help the readers better understand this method.**

We thank the reviewer for this suggestion. There are a number of differences between the assays, which is why we chose to include both measurements. First, arousal threshold is measured independent of time spent asleep (combining all sleeping flies at the time of stimulus) while reactivity specifically examines the response as a function of time asleep. Second, we believe there is a daytime ceiling effect in the figure previously labeled 2E. We have added additional details to the Results, the Figure Legend, as well as in the Methods (Lines 128, 139 and 450). Additionally, we now include day and nighttime sleep throughout the manuscript. We hope that this additional information allows the reader to identify specific and generalizable phenotypes.

**3. NF1 mutation and knockdown pan-neuronal increases waking activity (Fig S1B, S2D). Therefore, it is logical that metabolic rate is increased in these flies (Fig 3H-J). The important questions, that are not addressed in this study, are if the change in metabolic rate is due to the loss of deep sleep per se, and whether it is specifically regulated by NF1. For example, what if we compare VCO2 between awake period and sleep period for each genotype?**

We thank the reviewer for this suggestion. We find that measurements of metabolic rate are elevated during both waking and sleeping. This is consistent in *Nf1* mutants and upon pan-neuronal knockdown of Nf1, suggesting metabolic rate is regulated by *Nf1*. We now include these data in Supplemental Figure 6.

**4. Reduced longevity, intestinal barrier dysfunction, and increase in ROS levels in the gut by NF1 mutants and knockdown are interesting observations. But again, whether this is due to sleep loss per se, or specific to NF1 loss of function, are not addressed. This is especially important since NF1 is a well-known tumor suppressor.**

We acknowledge that *Nf1* is highly pleiotropic, which presents a challenge to address the causal nature of the phenotypes We acknowledge the limitations of these conclusions within the text. We now provide additional discussions of these potential interactions in the Discussion (Line 373).

**5. Are the number of GABA neurons similar between controls and Rdl/Nf1 KD flies? Could the effect observed in Fig.4 be due to a decrease in the number of GABA neurons?**

Thank you for this suggestion. We have now directly compared the expression pattern of Rdl-GAL4 expression within control and *Nf1*-knockdown flies. Qualitatively, we do not observe any differences in their expression patterns. In addition, we observe no change in overall fluorescence intensity between the *Nf1* deficient flies and their respective control. This data is now shown in Supplemental Fig 10 and described in the Results (Line 196).

**Minor comments**

**1. Line 40-41. ..., including a reduction in whole body metabolic rate (Refs.). Papers cited in this sentence do not support this statement.**

Thank you for identifying this error. We have now included the appropriate references, and cite Brown *et al* 2022 and Stahl *et al* 2017.

**2. Results, line- 87-108. These results reproduce the previous studies, the relevant literature should be cited, for example King et al. G3 (2016).**

We have now included citations to both Bai and Sehgal, 2015 and King et al., 2016.

**3. Please describe the number of flies used and the number of replicates performed.**

We now include sample size of each experiment in each figure legend. We also now state in the Methods: “A minimum of two independent runs were performed for each experiment, while the sample size for each genotype/treatment are presented in the figure legends” (Line 546).

**4. Why were just females used in the arousal threshold assay and for immunostaining whereas males were used in the other experiments?**

We thank the reviewer for catching this typo. Male flies were used for all experiments. We have made these changes in the text.

**Reviewer #3:**

**Giorgio Gilestro (note, Reviewer 3 has signed this review): The manuscript offers a very good phenotypical characterisation of Nf1 (mutants and RNAi) in Drosophila sleep. It reads well and is nicely illustrated. The figures are clear, the stats are well presented, the choice of plots is functional and rigorous. The introduction is well written and rather comprehensive. In general, I think the manuscript would be an excellent addition for people interested in fly models of disease and certainly sets a very high bar for that field.**

We thank the reviewer (Giorgio) for their supportive remarks. Indeed, we believe these studies build on the work of many groups within the field who have developed the *Nf1* model in *Drosophila* as well as tools for examining sleep depth. We hope this is conveyed throughout the manuscript.

**There is only part that did not convince me, as** **I think the conclusions are not supported by the data: the fact that the longevity and gut phenotype are due to lack of sleep. Nf1 is known to interfere with other aspects of behaviour. For instance, Bai et al 2018 showed a role for Nf1 in regulation of circadian rhythm and a role on hormone producing cells (DH44 and others). King et al 2016 showed that lack of Nf1 increases spontaneous grooming. Botero et al 2021 (in collaboration with this group) showed that the change in metabolic changes is not sleep specific. Moscato et al 2020 characterises a social behaviour phenotype. And the list could go on and on.**

Thank you for identifying this limitation in our initial manuscript. We agree that the results presented were correlative. We were particularly enthusiastic about the two primary phenotypes identified because they link an identified sleep quality phenotype with a recently identified role for gut dysregulation in sleep deprived flies (Vaccaro et al, 2020). We believe that as a general point, identifying causality amongst the many phenotypes in *Nf1* deficient animals is an enormous challenge. In the revised version, we provide a number of additional data points that support this link. First, we rule out *Rdl* expression in the gut as a cause of the phenotype (Supplemental Fig 9). Second, we find that dietary restriction increases lifespan and rescues these gut phenotypes in *Nf1* deficient flies (Fig 5). However, the effects of dietary restriction on sleep are complex, preventing us from directly linking them. Therefore, we believe we have provided additional data to support this link, though far from conclusive. We have toned down the conclusions of the manuscript and added a statement describing the limitations of data supporting our hypothesis (Line 373).

**Also, the n-syb line that the authors use for RNAi is not specific to the brain: Cognigni et al 2011 showed that enteric neurons strongly express n-syb and the modenseq data show very strong expression in the muscles too. So the gut phenotype could have in principle nothing to do with lack of sleep and I do not think that one can assume that the longevity and gut permeability effects are necessarily due to that - many other things are going on beside sleep loss!**

Thank you for highlighting this. We have performed a number of experiments to address this. First, we now provide imaging of the gut in *Rdl*-GAL4 flies with and without the GAL4 suppressor *tsh*-GAL80. Without *tsh*-GAL80, we find a small amount of GFP signal innervating the ampula of the intestine (Fig 3A). In the presence of *tsh*-GAL80, the sleep depth phenotype persists, and there is no gut expression (Supplemental Fig 9). We do not see obvious phenotypes elsewhere. Therefore, the phenotypes observed are likely neuronal. Further, we have attempted to examine the effects of longevity using the enteroblast driver Su(H)GBE-Gal4 (EB-GAL4; Zeng et al 2010). To our surprise, this led to a dramatic reduction in longevity. However, upon evaluation, this driver also labels many regions of the brain, some of which overlap with *Rdl*. While it would be possible to use intersectional techniques to develop more specific drivers (and we plan to do so). We prefer not to include these data in the manuscript because the results remain inconclusive at this time (**See Figure 2 below**).

**Figure 2. Knockdown of *Nf1* in enteroblasts reduces lifespan.** Enteroblasts (EBs) were targeted using the EB-GAL4 driver. **A.** The expression pattern of ECs is visualized with GFP in the brain (left) and the gut (right). For the brain, background staining is NC82 antibody (magenta); scale bar = 100μm. For the gut, background staining is DAPI (blue); scale bar = 1000μm. **B.** Compared to controls, knockdown of *Nf1* in EBs significantly decreases longevity (Log-Rank test: χ^2^=78.9, d.f.=2, *P*<0.0001). N=51-66.

**Also, we know that there are conditions in which lack of sleep does *not* lead to a decrease in longevity at all: eg. fumin mutants as in Kume et al 2005; natural short sleepers as in Harbison et al 2017; and mechanical sleep deprivation as in Geissmann et al 2019. So I think one should be way more cautious about these conclusions.**

We fully agree with this statement and have now added discussion of this, as well as the additional references. We now state in the Discussion: “However, there are many examples of short sleeping genetic mutants, as well as in artificially selected short-sleeping flies and chronically sleep deprived wildtype flies where lifespan is not impacted (Geissmann et al., 2019; Harbison et al., 2017; Kume et al., 2005; Stavropoulos & Young, 2011).” (Line 352).

**A shorter lifespan in Nf1 mutants was observed by Jiayuan Tong et al 2007 who also characterised this phenotype as linked to a defect in ROS production and it may be important to notice that: 1) they could rescue the phenotype by giving antioxidants in the diet and 2) they did not see a similarly strong phenotype with other mutant alleles, such as Nf1P2.**

Thank you for pointing this out. We now include discussion of these fundings (Line 382). In our hands, we do not see a robust impact of ROS or longevity with antioxidants (see response to Reviewer 2). We do not doubt these previous findings (or those of others) and instead believe the protocols require optimization in our hands. While we did not examine *Nf1*^P2^, we have verified this response with multiple RNAi lines, and using multiple different GAL4 drivers.

**If I were interested in pursuing that conclusion I would probably consider one of the following experiments:**

**- look at other Nf1 alleles or RNAi drivers. Can one find a condition that dissociates sleep loss from longevity/gut phenotype.**

**- feed NF1P1 flies with antioxidants. The hypothesis there is that one should be able to rescue the longevity phenotype but not the sleep loss (as shown already in Vaccaro et al).**

**- Drive Nf1 RNAi only in the gut. This should be an easy experiment and would be my first step.**

Thank you for these additional suggestions. We are currently pursuing them, or optimizing the data as described above. We have included a final paragraph that is forward looking in the hopes of highlighting the future potential of this work (Line 402). We also note that we have added one set of experiments (dietary restriction) that fully rescues the gut phenotypes (Fig 5), though we also note that these data unlock additional questions.
