# Supplementary material for: Neurofibromin 1 mediates sleep depth in Drosophila
Source: PLoS Genet. 2023 Dec 13;19(12):e1011049. doi: 10.1371/journal.pgen.1011049 (PMC10763969; doi:10.1371/journal.pgen.1011049)
Supplement: Attachment — Submitted filename: Reviewer Comments R2.docx [file pgen.1011049.s017.docx]

We are grateful for the reviewers comments and their attention to multiple rounds of this manuscript. We hope to have addressed them with the changes described below.

**Reviewer #2:**

**The author have addressed all the minor points reviewers made adding to both the figures and supplementary figures. They added to the text references to previous papers that had shown very similar results to place the work in context. The manuscript is strengthen and slightly more cautious in the discussion (but not so much in the abstract).**

**Here are some specific comments:**

We thank the reviewer for their thoughtful feedback. We’ve made additional edits to the abstract to further tone down our conclusions. We agree that we had overinterpreted our data, and hope the current framing accurately reflects some limitations of our data.

**# Convincing points:**

**(Fig 1-2) I would agree that their work is novel in further demonstrating the NF1 sleep phenotype, expanding it beyond just a decrease in sleep and accurately characterising it. Their evidence for disrupted depth of sleep is convincing, sleep is shown to be fragmented (especially at night) with a similar p(doze) value during the night whilst having an increased p(wake) value, demonstrating sleep pressure is still relatively normal but with an abnormal wake pressure, disrupting sleep at an early stage. Additionally the metabolic rate increase is particularly convincing with both mutants and the RNAi line only decreasing slightly over 40 minutes, showing they lack a signature of deep sleep. Maybe this shows that the process of metabolic down scaling is an integral part of the transition to deep sleep in drosophila.**

We have added to the discussion: “While it is possible that the lack of metabolic downscaling during sleep bouts is due to a lack of deep sleep, it is also possible that metabolic downscaling itself is required for deep sleep” (Line 320).

**(Supp fig 9) They show the phenotype of null Nf1 is brain specific to the Rdl expessing GABA neurons with tsh-GAL80 - addressing the n-syb brain/gut expression problem.**

**(Supp fig 13) Rescue experiments with Gaboxadol failed to rescue the gut or longevity phenotype. This is interesting as gaboxadol is thought to induce a SWS like state (Anthoney et al 2023), however here whilst increasing sleep the arousal threshold remained low (it increased it in controls). It would be nice to see the metabolic rate too to further understand if flies are not entering a deeper sleep. This could further suggest that Nf1 is integral for the transition to a deep sleep state, blocking the ability of gaboxadol to rescue deep sleep in this case.**

We agree. We are interested in following up this work investigating the signaling pathways downstream of *Nf1* and how they may regulate metabolic rate during deep sleep.	This is very time consuming, and we are working to reestablish the system (with improved sensitivity) since the lab has moved. We hope to perform these experiments as part of future work.

**# Confusing or not convincing:
(Fig 5) I find the dietary restriction conclusions to be confusing. There are some important aspects to address here, I think:
1- It is not clear if the ab libitum (AL) diet (which they describe as high protein) is the standard media used throughout. If it’s different then they should also plot the standard media.**

The ad libitum diet used in this set of experiments was derived from the Caltech recipe, whereas the standard food used in all other experiments was derived from the Bloomington recipe. The Caltech recipe was used to emulate previous work that showed DR extends lifespan (Akagi et al 2018). We have changed *Ad libitum* to standard diet, which more accurately describes the protocol used.

**2 - The survival curve for the dietary restriction (DR) longevity in Figure 5 is very similar (~ 50 days) to the Rdl NF1 plot in figure 4 (with AL being a lot less than both at ~ 30 days).
If AL is considered the normal conditions, what counts for this discrepancy?**

We think this discrepancy arises from the difference between the Caltech and Bloomington fly food recipes. We have added a statement in the Methods that addresses this. We now state: The Caltech standard food diet is different from the Bloomington standard food diet used in the experiments described above, which differ in their protein:carbohydrate ratio (Lesperance and Broderick 2020)” (Line 529).

**3 - The authors show that in DR sleep time is still reduced (Figure 5E) with lower arousal threshold (Fig 5F) and high night reactivity (Fig 5H), which the authors have continually stated is the hallmark of lack of deep sleep. So the flies are still not deep sleeping, they have normal ROS, and display a short living phenotype. Together with the finding that antioxidants are not affecting longevity, this clearly indicates that the longevity phenotype it’s independent of ROS, doesn't it?**

We agree. To further support these findings, we now include the antioxidant feeding data as Supplementary Figure 13. We state in the results: “To test whether antioxidant feeding in *Nf1*^RNAi^ flies would promote survival and restore gut homeostasis, we added either Lipoic Acid or Melatonin to standard *Drosophila* media and then measured longevity and ROS in the gut. We found that antioxidant feeding had no effect on longevity, intestinal permeability, or ROS in Rdl-GAL4/*Nf1*^RNAi^ flies (Fig S13). Overall, these findings support the notion that reduced gut function contributes to the reduced lifespan associated with the loss of *Nf1* and suggests that *Nf1* may act upstream of the protective effects of these antioxidants on lifespan and gut function.” (Line 243).’ Our current sense is that the overall effects of anti-oxidants are insufficiently robust to draw strong conclusions. We hope that including these data in our supplemental data will allow others to build on this work.

**To conclude: I am in favor of publishing this paper and find the links between Nf1 and sleep depth & metabolic rate convincing, but I would certainly come to the conclusion that the longevity phenotype may be confounded by some other action of NF1 as the results show quite clearly neither sleep nor ROS are actually linked to it.**

Again, we thank the reviewer for their feedback. We hope we have sufficiently addressed the concerns noted above.
