# Supplementary material for: Neurofibromin 1 mediates sleep depth in Drosophila
Source: PLoS Genet. 2023 Dec 13;19(12):e1011049. doi: 10.1371/journal.pgen.1011049 (PMC10763969; doi:10.1371/journal.pgen.1011049)
Supplement: Peer review history

# Author comment: Author response to previous submission

**DOI:** 10.1371/journal.pgen.1011049.r001

**Transfer Alert**

This paper was transferred from another journal. As a result, its full editorial history (including decision letters, peer reviews and author responses) may not be present.

11 Sep 2023

Attachment Submitted filename: Reviewer Comments V4.docx Click here for additional data file.

# Peer review: Decision Letter 0

**DOI:** 10.1371/journal.pgen.1011049.r002

**Authors:** Gregory S. Barsh

28 Sep 2023

Dear Dr Keene,

Thank you very much for submitting your Research Article entitled 'Neurofibromin 1 mediates sleep depth in Drosophila' to PLOS Genetics.

The manuscript was seen by 2 of the 3 previous reviewers at PLOS Biology. As you will see, both reviewers are positive; however, there are some remaining concerns that we ask you address in a revised manuscript.

We therefore ask you to modify the manuscript according to the review recommendations. Your revisions should address the specific points made by each reviewer.

We hope to receive your revised manuscript within the next 30 days. If you anticipate any delay in its return, we would ask you to let us know the expected resubmission date by email to plosgenetics@plos.org.

If present, accompanying reviewer attachments should be included with this email; please notify the journal office if any appear to be missing. They will also be available for download from the link below. You can use this link to log into the system when you are ready to submit a revised version, having first consulted our Submission Checklist.

While revising your submission, please upload your figure files to the Preflight Analysis and Conversion Engine (PACE) digital diagnostic tool. PACE helps ensure that figures meet PLOS requirements. To use PACE, you must first register as a user. Then, login and navigate to the UPLOAD tab, where you will find detailed instructions on how to use the tool. If you encounter any issues or have any questions when using PACE, please email us at figures@plos.org.

Please be aware that our data availability policy requires that all numerical data underlying graphs or summary statistics are included with the submission, and you will need to provide this upon resubmission if not already present. In addition, we do not permit the inclusion of phrases such as "data not shown" or "unpublished results" in manuscripts. All points should be backed up by data provided with the submission.

To enhance the reproducibility of your results, we recommend that you deposit your laboratory protocols in protocols.io, where a protocol can be assigned its own identifier (DOI) such that it can be cited independently in the future. Additionally, PLOS ONE offers an option to publish peer-reviewed clinical study protocols. Read more information on sharing protocols at https://plos.org/protocols?utm_medium=editorial-email&utm_source=authorletters&utm_campaign=protocols

Please review your reference list to ensure that it is complete and correct. If you have cited papers that have been retracted, please include the rationale for doing so in the manuscript text, or remove these references and replace them with relevant current references. Any changes to the reference list should be mentioned in the rebuttal letter that accompanies your revised manuscript. If you need to cite a retracted article, indicate the article’s retracted status in the References list and also include a citation and full reference for the retraction notice.

PLOS has incorporated Similarity Check, powered by iThenticate, into its journal-wide submission system in order to screen submitted content for originality before publication. Each PLOS journal undertakes screening on a proportion of submitted articles. You will be contacted if needed following the screening process.

To resubmit, you will need to go to the link below and 'Revise Submission' in the 'Submissions Needing Revision' folder.

Please let us know if you have any questions while making these revisions.

Yours sincerely,

Gregory S. Barsh

Editor-in-Chief

PLOS Genetics

Gregory Copenhaver

Editor-in-Chief

PLOS Genetics

Reviewer's Responses to Questions

**Comments to the Authors:**

**Please note here if the review is uploaded as an attachment.**

Reviewer #1: The revised manuscript addresses my previous comments, I have no new suggestions to raise.

Reviewer #2: The author have addressed all the minor points reviewers made adding to both the figures and supplementary figures. They added to the text references to previous papers that had shown very similar results to place the work in context. The manuscript is strengthen and slightly more cautious in the discussion (but not so much in the abstract). Here are some specific comments:

# Convincing points:

(Fig 1-2) I would agree that their work is novel in further demonstrating the NF1 sleep phenotype, expanding it beyond just a decrease in sleep and accurately characterising it. Their evidence for disrupted depth of sleep is convincing, sleep is shown to be fragmented (especially at night) with a similar p(doze) value during the night whilst having an increased p(wake) value, demonstrating sleep pressure is still relatively normal but with an abnormal wake pressure, disrupting sleep at an early stage. Additionally the metabolic rate increase is particularly convincing with both mutants and the RNAi line only decreasing slightly over 40 minutes, showing they lack a signature of deep sleep. Maybe this shows that the process of metabolic down scaling is an integral part of the transition to deep sleep in drosophila.

(Supp fig 9) They show the phenotype of null Nf1 is brain specific to the Rdl expessing GABA neurons with tsh-GAL80 - addressing the n-syb brain/gut expression problem

(Supp fig 13) Rescue experiments with Gaboxadol failed to rescue the gut or longevity phenotype. This is interesting as gaboxadol is thought to induce a SWS like state (Anthoney et al 2023), however here whilst increasing sleep the arousal threshold remained low (it increased it in controls). It would be nice to see the metabolic rate too to further understand if flies are not entering a deeper sleep. This could further suggest that Nf1 is integral for the transition to a deep sleep state, blocking the ability of gaboxadol to rescue deep sleep in this case.

# Confusing or not convincing:

(Fig 5) I find the dietary restriction conclusions to be confusing. There are some important aspects to address here, I think:

1- It is not clear if the ab libitum (AL) diet (which they describe as high protein) is the standard media used throughout. If it’s different then they should also plot the standard media.

2 - The survival curve for the dietary restriction (DR) longevity in Figure 5 is very similar (~ 50 days) to the Rdl NF1 plot in figure 4 (with AL being a lot less than both at ~ 30 days).

If AL is considered the normal conditions, what counts for this discrepancy?

3 - The authors show that in DR sleep time is still reduced (Figure 5E) with lower arousal threshold (Fig 5F) and high night reactivity (Fig 5H), which the authors have continually stated is the hallmark of lack of deep sleep. So the flies are still not deep sleeping, they have normal ROS, and display a short living phenotype. Together with the finding that antioxidants are not affecting longevity, this clearly indicates that the longevity phenotype it’s independent of ROS, doesn't it?

To conclude: I am in favour of publishing this paper and find the links between Nf1 and sleep depth & metabolic rate convincing, but I would certainly come to the conclusion that the longevity phenotype may be confounded by some other action of NF1 as the results show quite clearly neither sleep nor ROS are actually linked to it.

**********

**Have all data underlying the figures and results presented in the manuscript been provided?**

Large-scale datasets should be made available via a public repository as described in the *PLOS Genetics* data availability policy, and numerical data that underlies graphs or summary statistics should be provided in spreadsheet form as supporting information.

Reviewer #1: None

Reviewer #2: Yes

**********

PLOS authors have the option to publish the peer review history of their article (what does this mean?). If published, this will include your full peer review and any attached files.

If you choose “no”, your identity will remain anonymous but your review may still be made public.

**Do you want your identity to be public for this peer review?** For information about this choice, including consent withdrawal, please see our Privacy Policy.

Reviewer #1: No

Reviewer #2: **Yes: **Giorgio Gilestro

# Author comment: Author response to Decision Letter 0

**DOI:** 10.1371/journal.pgen.1011049.r003

28 Oct 2023

Attachment Submitted filename: Reviewer Comments R2.docx Click here for additional data file.

# Editor report: Decision Letter 1

**DOI:** 10.1371/journal.pgen.1011049.r004

**Authors:** Gregory S. Barsh

3 Nov 2023

Dear Dr Keene,

We are pleased to inform you that your manuscript entitled "Neurofibromin 1 mediates sleep depth in Drosophila" has been editorially accepted for publication in PLOS Genetics. Congratulations!

Before your submission can be formally accepted and sent to production you will need to complete our formatting changes, which you will receive in a follow up email. Please be aware that it may take several days for you to receive this email; during this time no action is required by you. Please note: the accept date on your published article will reflect the date of this provisional acceptance, but your manuscript will not be scheduled for publication until the required changes have been made.

Once your paper is formally accepted, an uncorrected proof of your manuscript will be published online ahead of the final version, unless you’ve already opted out via the online submission form. If, for any reason, you do not want an earlier version of your manuscript published online or are unsure if you have already indicated as such, please let the journal staff know immediately at plosgenetics@plos.org.

In the meantime, please log into Editorial Manager at https://www.editorialmanager.com/pgenetics/, click the "Update My Information" link at the top of the page, and update your user information to ensure an efficient production and billing process. Note that PLOS requires an ORCID iD for all corresponding authors. Therefore, please ensure that you have an ORCID iD and that it is validated in Editorial Manager. To do this, go to ‘Update my Information’ (in the upper left-hand corner of the main menu), and click on the Fetch/Validate link next to the ORCID field.  This will take you to the ORCID site and allow you to create a new iD or authenticate a pre-existing iD in Editorial Manager.

If you have a press-related query, or would like to know about making your underlying data available (as you will be aware, this is required for publication), please see the end of this email. If your institution or institutions have a press office, please notify them about your upcoming article at this point, to enable them to help maximise its impact. Inform journal staff as soon as possible if you are preparing a press release for your article and need a publication date.

Thank you again for supporting open-access publishing; we are looking forward to publishing your work in PLOS Genetics!

Yours sincerely,

Gregory S. Barsh

Editor-in-Chief

PLOS Genetics

Gregory Copenhaver

Editor-in-Chief

PLOS Genetics

www.plosgenetics.org

Twitter: @PLOSGenetics

----------------------------------------------------

Comments from the reviewers (if applicable):

----------------------------------------------------

**Data Deposition**

If you have submitted a Research Article or Front Matter that has associated data that are not suitable for deposition in a subject-specific public repository (such as GenBank or ArrayExpress), one way to make that data available is to deposit it in the Dryad Digital Repository. As you may recall, we ask all authors to agree to make data available; this is one way to achieve that. A full list of recommended repositories can be found on our website.

The following link will take you to the Dryad record for your article, so you won't have to re‐enter its bibliographic information, and can upload your files directly: 

http://datadryad.org/submit?journalID=pgenetics&manu=PGENETICS-D-23-00920R1

More information about depositing data in Dryad is available at http://www.datadryad.org/depositing. If you experience any difficulties in submitting your data, please contact help@datadryad.org for support.

Additionally, please be aware that our data availability policy requires that all numerical data underlying display items are included with the submission, and you will need to provide this before we can formally accept your manuscript, if not already present.

----------------------------------------------------

**Press Queries**

If you or your institution will be preparing press materials for this manuscript, or if you need to know your paper's publication date for media purposes, please inform the journal staff as soon as possible so that your submission can be scheduled accordingly. Your manuscript will remain under a strict press embargo until the publication date and time. This means an early version of your manuscript will not be published ahead of your final version. PLOS Genetics may also choose to issue a press release for your article. If there's anything the journal should know or you'd like more information, please get in touch via plosgenetics@plos.org.

# Editor report: Acceptance letter

**DOI:** 10.1371/journal.pgen.1011049.r005

**Authors:** Gregory S. Barsh

29 Nov 2023

PGENETICS-D-23-00920R1 

Neurofibromin 1 mediates sleep depth in Drosophila 

Dear Dr Keene, 

We are pleased to inform you that your manuscript entitled "Neurofibromin 1 mediates sleep depth in Drosophila" has been formally accepted for publication in PLOS Genetics! Your manuscript is now with our production department and you will be notified of the publication date in due course.

The corresponding author will soon be receiving a typeset proof for review, to ensure errors have not been introduced during production. Please review the PDF proof of your manuscript carefully, as this is the last chance to correct any errors. Please note that major changes, or those which affect the scientific understanding of the work, will likely cause delays to the publication date of your manuscript. 

Soon after your final files are uploaded, unless you have opted out or your manuscript is a front-matter piece, the early version of your manuscript will be published online. The date of the early version will be your article's publication date. The final article will be published to the same URL, and all versions of the paper will be accessible to readers.

Thank you again for supporting PLOS Genetics and open-access publishing. We are looking forward to publishing your work! 

With kind regards,

Zsofi Zombor

PLOS Genetics

On behalf of:

The PLOS Genetics Team

Carlyle House, Carlyle Road, Cambridge CB4 3DN | United Kingdom

plosgenetics@plos.org | +44 (0) 1223-442823

plosgenetics.org | Twitter: @PLOSGenetics
